# TRP channel expression patterns define molecular subtypes, prognosis, and therapeutic targets in gastric cancer

**DOI:** 10.3389/fimmu.2026.1752001

**Published:** 2026-03-05

**Authors:** Zheng Ding, Wei Liu, Zi-xi Li, Jian-qing Cheng, Chao Deng, Ke-wei Wang, Ling-jun Li

**Affiliations:** 1 Institute of Integrated Traditional Chinese and Western Medicine, Affiliated Hospital of Jiangnan University, Wuxi, China; 2 Department of General Surgery, Jiangnan University Medical Center, Wuxi, China; 3 Wuxi School of Medicine, Jiangnan University, Wuxi, China; 4 Department of Ultrasound, Affiliated Wuxi Chinese Medicine Hospital, Nanjing University of Chinese Medicine, Wuxi, Jiangsu, China

**Keywords:** gastric cancer, molecular subtypes, prognosis, therapeutic targets, TRP channel

## Introduction

Gastric cancer (GC) ranks as the fifth most prevalent malignancy and fourth leading cause of cancer mortality worldwide, with highest incidence rates observed in Asia (1, 2). While typically asymptomatic in early stages, diagnosis at advanced or metastatic phases relies primarily on endoscopic examination and biopsy (3). Current treatments for late-stage disease, including chemotherapy and combination surgery, demonstrate variable efficacy due to tumor heterogeneity, often resulting in poor prognosis. Although emerging targeted therapies and immunotherapies show promise by leveraging tumor expression profiles and genomic characteristics (4, 5), significant interpatient variability and limited therapeutic targets remain substantial challenges.

The transient receptor potential (TRP) channel family has become the focus of attention in cancer research because of its essential functions in cell growth and survival pathways (6). These channels promote cancer progression by modulating Ca2^+^ signaling, which affects proliferation, apoptosis, transcriptional regulation, and angiogenesis (7). In GC, various TRP isoforms such as TRPM2, TRPV4, and TRPC1/3/6 have shown oncogenic significance; nevertheless, their overall contribution to GC pathogenesis is not fully elucidated (8–10). Prior research has predominantly focused on individual components, resulting in an inadequate understanding of the collective expression patterns, co-regulatory interactions, and systemic effects of TRP channels in this malignancy.

**Figure 1:**
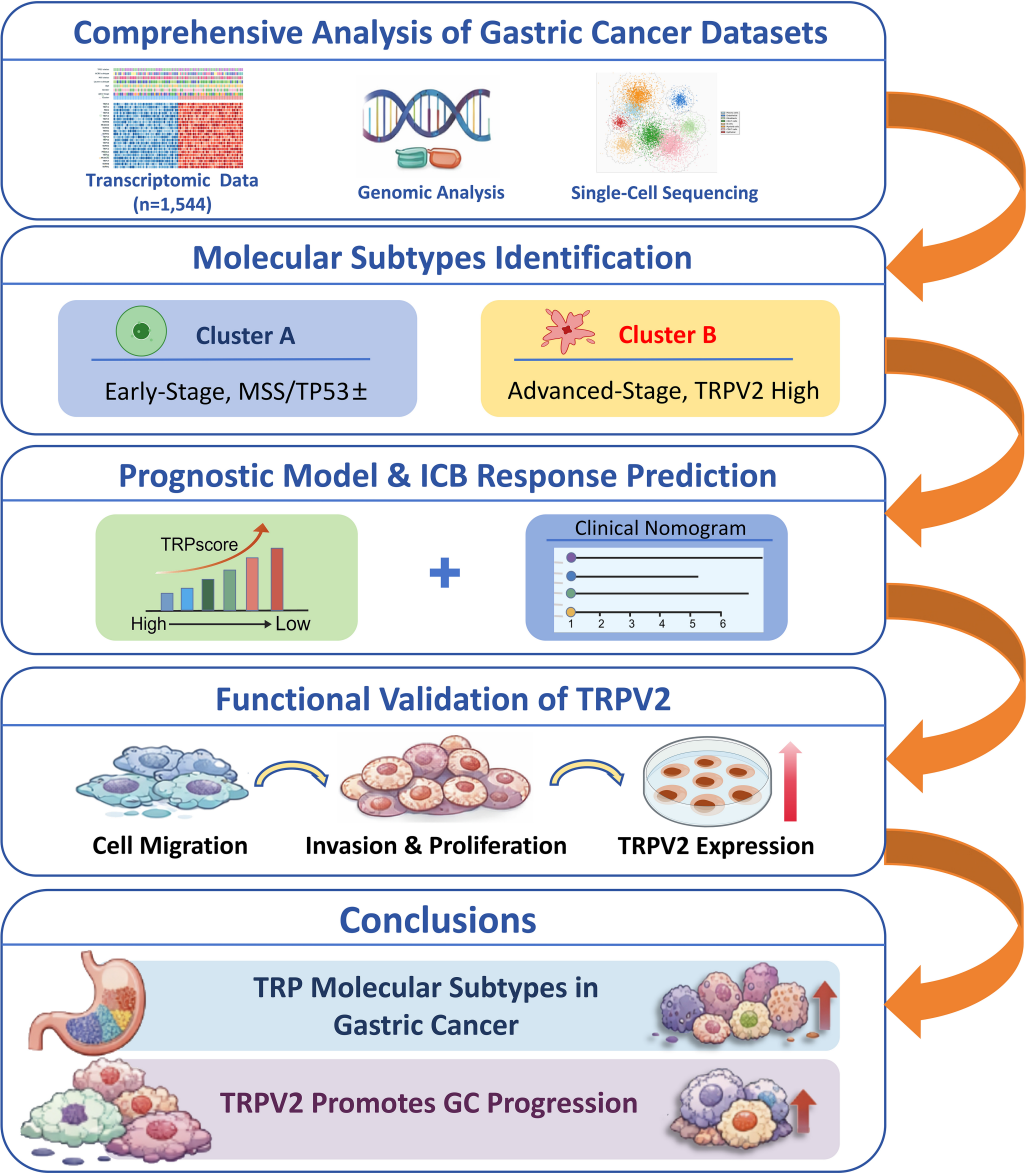
Study workflow and analytical pipeline.

Here, we combined seven GC datasets from the Cancer Genome Atlas (TCGA) cohort and six Gene Expression Omnibus (GEO) groups (11–16). Different TRP subtypes were found using unsupervised grouping based on TRP channel regulatory factors. We created the TRPscore using differentially expressed genes (DEGs) between subtypes to measure prognostic value and guess the makeup of the tumor microenvironment (TME) and the response to immunotherapy. We created a full nomogram using TRPscore and other molecular features to estimate each individual’s perspective. Single-cell transcriptomic (scRNA-seq) analysis showed that TRPV2 was mostly expressed in certain types of cells (17). Functional confirmation showed that TRPV2 was an essential element for assisting GC cells move, invade, and proliferate *in vitro*. The workflow of our research is shown in **Figure 1**, showing our five main study phases: multi-cohort data integration, TRP-based molecular subtyping (Cluster A/B), TRPscore building and validation for prognosis and immunotherapy prediction, TRPV2 functional validation, and precision medicine clinical implications.

## Materials and methods

### Patients and data collection

We obtained gene expression data from the GEO (https://www.ncbi.nlm.nih.gov/geo) and TCGA (http://xena.ucsc.edu/) databases, excluding GC patients lacking survival information. The final analysis comprised six GEO datasets—GSE15459 (n=200), GSE29272 (n=134), GSE34942 (n=56), GSE57303 (n=70), GSE62254 (n=300), and GSE84437 (n=433)—along with TCGA-STAD. After excluding samples without survival data, these were consolidated into the STAD-Cohort (n=1,544). For TCGA data, RNA sequencing (FPKM values) was retrieved using the R package TCGAbiolinks and converted to transcripts per kilobase million (TPM) (18). GEO datasets were processed using the ArrayExpress package (19). Batch effects were corrected via the sva package’ s “ComBat” algorithm (20). Additionally, scRNA-seq data (GSE206785) was downloaded from GEO and preprocessed using Seurat (21).

### Analysis of mutation and copy number difference

The R package ‘maftools’ was utilized to generate waterfall plots depicting gene mutations and copy number variations within the TCGA-STAD cohort. For copy number analysis across TCGA pan-cancer datasets, the GSCALite web portal (https://guolab.wchscu.cn/GSCA/#/) was employed to identify genomic amplifications and gene deletions. Copy number gains or losses were determined based on the aggregate count of genes exhibiting copy number alterations at both focal and chromosomal arm levels.

### TRP subtypes construction

Originally, 28 TRP channel regulators were identified. After integrating seven datasets, 22 common TRP channel regulators (TRPA1, TRPM1-8, TRPV1-6, TRPC2-7) were selected for analysis. Unsupervised clustering using the ConsensusClusterPlus R package (22) was performed on all samples based on these 22 regulators expression levels. The optimal number of clusters was determined to be two, supported by stability assessment. Principal component analysis (PCA) was used to confirm this two-cluster classification. To better understand the different characteristics of TRP subtypes of GC. We compared the relationship between the two subtypes and several known GC types, including the Asian cancer research group (ACRG) and Lauren subtypes. The R packages ‘TCGAbiolinks’ and ‘CancerSubtypes’ were used to identify the subtypes of GC in the GSE62254 cohort (23).

### TME in GC

Differential expression of key tumor immune-related genes was compared across TRP subgroups using the limma package (24). Immune cell infiltration levels in GC were quantified through six established algorithms: TIMER, QUANTISEQ, MCPcounter, EPIC, CIBERSORT, and xCell (25). Subsequently, single-sample gene set enrichment analysis (ssGSEA) was performed to evaluate differences in immune cell infiltration and immune function scores between TRP subtypes and 22 TRP channel-regulated factors (26).

### Identification of DEGs

The limma package implemented an empirical Bayesian approach to detect DEGs among TRP clusters, with false discovery rate (FDR)-adjusted *P* < 0.05 considered significant. Subsequent functional annotation of DEGs was performed using ClusterProfiler for Gene Ontology (GO) and Kyoto Encyclopedia of Genes and Genomes (KEGG) analyses.

### Construction of TRPscore system

Weighted gene co-expression network analysis (WGCNA) constructed co-expression networks, from which module genes were selected to establish the TRPscore via ssGSEA. This scoring system was evaluated for its prognostic predictive capacity across GC subtypes and potential utility in immunotherapy response assessment. WGCNA analysis was performed using the R software “WGCNA” package (27).

### Prediction of the response to anti-PD-1 immunotherapy

A nomogram was developed based on multivariate Cox regression analysis incorporating key signaling pathways relevant to GC pathogenesis and TRPscore. The model was constructed using the ‘rms’ package in R. Model performance was assessed using calibration curves and receiver operating characteristic (ROC) curve analysis.

### scRNA-seq analysis

To determine whether TRP channel regulators is primarily expressed in specific cell populations, the GC scRNA-seq dataset GSE206785 was analyzed using the Seurat package in R (28). Low-quality cells (nFeature <1000 or mitochondrial gene percentage >10%) and potential duplexes (>6000 genes detected) were excluded. Data were normalized using the LogNormalize method, and the top 2000 highly variable genes were identified. Dimensionality reduction was performed via principal component analysis. Cell clusters were identified (resolution=0.5) and visualized using t-distributed stochastic neighbor embedding (t-SNE) Cluster-specific marker genes were determined and annotated using the CellMarker database (29).

### Cell culture, antibodies, and other materials

Human GC cell lines HGC27 and AGS were obtained from the Cell Bank of the Shanghai Institute of Biochemistry and Cell Biology, Chinese Academy of Sciences. Cells were maintained in high-glucose DMEM supplemented with 1% penicillin-streptomycin solution and 10% fetal bovine serum (FBS), under conditions of 37 °C and 5% CO_2_. Primary antibodies used included a rabbit polyclonal anti-TRPV2 antibody (BOSTER; dilution 1:1000), HRP-labeled streptavidin (Sangon Biotech; dilution 1:7000), an anti-GAPDH mouse monoclonal antibody (Sangon Biotech; dilution 1:2000), and HRP-conjugated goat anti-mouse IgG (Sangon Biotech; dilution 1:10,000).

### Quantitative real-time PCR

Total RNA was extracted using the RNAeasy™ Animal RNA Isolation Kit with Spin Column (Beyotime). First-strand cDNA synthesis was performed using the High-Capacity cDNA Reverse Transcription Kit (Thermo Fisher Scientific). mRNA expression levels were quantified via real-time PCR using UltraSYBR Mixture (CWBIO) Primer sequences are detailed in **Supplementary Table 12**.

### Western blotting

Cells were lysed in RIPA buffer supplemented with phenylmethanesulfonyl fluoride (PMSF). Protein concentrations were determined using the Enhanced BCA Protein Assay Kit (Beyotime). Equal amounts of protein were separated by SDS-PAGE (Vazyme Biotech Co., Ltd.) and transferred onto PVDF membranes (Merck Millipore). Membranes were incubated with primary antibodies, followed by appropriate secondary antibodies, and protein signals were detected using BeyoECL Plus (Beyotime).

### Small-interfering RNA transfection

Cells were transfected with 5 nmol/L siRNA (GENCEFE Biotech) using Lipo8000™ Transfection Reagent (Beyotime), following the manufacturer’s instructions. A non-targeting siRNA was used as a negative control. The sequences of si-TRPV2 are listed in **Supplementary Table 12**.

### Wound healing assay

For wound healing assays, cells transfected with either TRPV2-specific or control siRNA were cultured to near confluence. A scratch was created using a pipette tip, and detached cells were removed by washing with fresh medium. Wound closure was monitored every 12 hours using ImageJ software to quantify the cell-free area until complete closure was achieved.

### Transwell migration and invasion assay

For migration and invasion analyses, cells (20,000 for migration, 50,000 for invasion) were seeded into the upper chamber of Transwell inserts in serum-free medium. The lower chamber contained medium supplemented with 20% FBS. For invasion assays, Matrigel Matrix (BD Biocoat) was applied to the insert membrane. After incubation at 37 °C for 24–48 hours, non-migratory cells were removed from the upper surface. Migrated cells on the lower surface were fixed with 4% paraformaldehyde and stained with crystal violet (0.1%). Images of five random fields per insert were captured at 100× magnification, and cell numbers were quantified using ImageJ software.

### Colony formation assay

Cells were seeded at a density of 1,000 per well in 6-well plates and allowed to grow for 1–2 weeks. Colonies were then fixed with 4% paraformaldehyde, stained with crystal violet, and counted using ImageJ software.

### Establishment and validation of the nomogram

A nomogram was developed based on multivariate Cox regression analysis incorporating key signaling pathways relevant to GC pathogenesis and TRPscore. The model was constructed using the ‘rms’ package in R. Model performance was assessed using calibration curves and receiver operating characteristic (ROC) curve analysis.

### TRP score

The 1,071 genes in the turquoise module exhibited strong intercorrelation. To capture the most representative information from these expression profiles, we applied a dimensionality reduction strategy termed “consolidation,” as previously described (30). This approach condenses highly correlated variables into a smaller set of principal components while retaining the majority of the original variance. Briefly, principal component analysis (PCA) was performed on the gene expression matrix of the turquoise module (24). The first and second principal components (PC1 and PC2), which account for the largest fractions of variance, were extracted for each sample. The TRPscore was then calculated as the sum of PC1 and PC2:

$\text{TRP score}=\sum (\text{PC}1_{\text{i}}+\text{PC}2_{\text{i}})$where i represents the expression of each individual gene.

### Statistics

Statistical analyses were performed using appropriate methods based on data distribution and study design. For two-group comparisons, unpaired t-tests or Wilcoxon rank-sum tests were used for normally or non-normally distributed data, respectively. Multi-group comparisons employed one-way analysis of variance (ANOVA) or Kruskal-Wallis tests as appropriate. Correlation analyses utilized Pearson or Spearman methods. Survival curves were constructed using Kaplan-Meier estimation with log-rank testing for significance. Univariate and multivariate Cox proportional hazards models calculated hazard ratios (HRs) and 95% confidence intervals (CIs). WGCNA was conducted using the dedicated R package. Optimal TRPscore cutoff values for dichotomization were determined using the “Surv_cutpoint” function in Survminer. Multiple testing correction applied FDR adjustment. Statistical significance was set at *P* < 0.05. Data visualization and statistical computations were performed using GraphPad Prism 8 and R version 3.6.1, respectively.

## Results

### Genetic and transcriptional variations of TRP channel regulators

**Figure 2:**
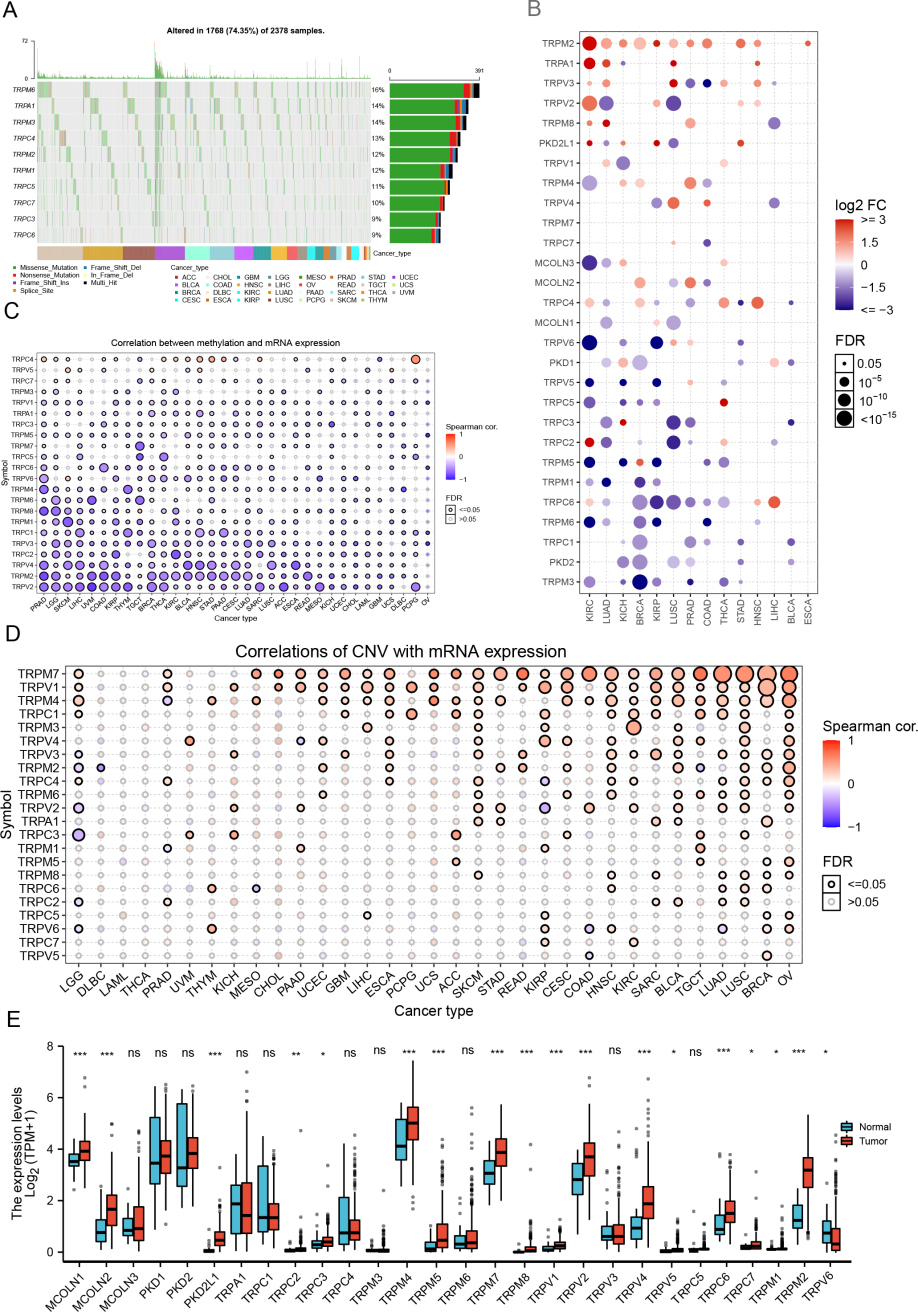
Genetic and epigenetic alterations of TRP channel regulators across human cancers. **(A)** Oncoplot displays the single nucleotide variant (SNV) landscape for the top 10 mutated genes among the input gene set in specific cancers. Among 2378 samples with at least one mutation in the 28 channel regulators, 1768 samples had at least one mutation in the top 10 genes, accounting for 74.35%. The percentage on the right side of the plot represents the ratio of samples with mutations in the corresponding gene to the total 2378 samples with at least one mutation in the 28 TRP channel regulators. **(B)** Dot plots illustrating expression differences of 28 TRP channel regulators between tumor and adjacent normal tissues across multiple cancer types in the TCGA pan-cancer dataset. The color of the dots reflects the degree of expression change; redder dots indicate higher expression in tumor tissues, while bluer dots indicate higher expression in normal tissues. Statistical significance was assessed using an adjusted t-test and FDR correction. Fold change was calculated as mean (Tumor)/mean (Normal). Bubble size represents FDR, with larger bubbles indicating lower FDR values. Only genes with fold changes greater than 2 and FDR ≤ 0.05 were included in the figure. Cancer types without significant genes were excluded from the final visualization. **(C)** The bubble plot illustrating the correlation between methylation levels and mRNA expression of the 28 TRP channel regulators. Bubble color indicates the strength of the correlation, with darker colors representing stronger correlations. Red denotes positive correlation, while blue denotes negative correlation. **(D)** The bubble plot depicts the correlation between copy number variation (CNV) and mRNA expression levels of the 28 TRP channel regulators. Bubble color reflects the strength of the correlation, with darker colors indicating stronger correlations. Red signifies positive correlation, and blue signifies negative correlation. Solid-line bubbles correspond to FDR ≤ 0.05. **(E)** Differences in mRNA expression of the 28 TRP channel regulators between normal and tumor tissues in TCGA-STAD were analyzed using the Wilcoxon test. The upper and lower ends of the box represent the interquartile range, the line within the box represents the median, and black dots represent outliers. Asterisks denote statistical significance; *P < 0.05, **P < 0.01, ***P < 0.001, ns, no significant.

We analyzed 28 TRP channel regulators (TRPC1–7, TRPV1–6, TRPM1–8, TRPP1–3, TRPML1–3, TRPA1) across 2378 pan-TCGA samples with mutations in at least one regulator. Among these, 1768 (74.35%) harbored TRP mutations, with TRPM6 (16%), TRPA1 (14%), and TRPM3 (14%) exhibiting the highest frequencies. Missense mutations predominated, and melanoma (TCGA-SKCM) showed the highest mutation burden (**Figures 2A**, **Supplementary Table 1**).

Most TRP regulators displayed lower mRNA expression in tumors, except TRPM2, TRPA1, TRPV3, TRPV2, TRPM8, PKD2L1, TRPV1, and TRPM4 (**Figure 2B**). Methylation was significantly downregulated in tumors for most regulators, except TRPC5, TRPC6, TRPC3, TRPA1, TRPC1, TRPV4, TRPM3, PKD1, and MCOLN3 (**Supplementary Figure 1A**). Methylation and mRNA expression were inversely correlated, particularly for TRPM2 and TRPV2 (**Figure 2C**), while copy number variation (CNV) and mRNA levels were positively correlated, notably for TRPM7 (**Figure 2D**).

CNV alterations were widespread, with TRPA1, TRPV5, and TRPV6 most frequently affected, primarily via heterozygous amplification or loss of heterozygosity; homozygous CNV was rare (**Supplementary Figures 1B–E**). In addition, we also presented the CNV sites of TRP channel regulatory factors on 23 pairs of human chromosomes (**Supplementary Figure 1D**). Furthermore, elevated TRP regulator expression conferred a survival advantage in several cancers (**Supplementary Figure 1F**). Heterogeneous expression patterns between normal and tumor tissues suggested that dysregulation correlates with genomic instability. Co-expression patterns among certain regulators imply cooperative roles in tumorigenesis, warranting further mechanistic studies to elucidate their therapeutic potential.

### TRP channel regulators define molecular subtypes with prognostic significance

Based on prior evidence (6), we further investigated TRP channel regulators in GC using the TCGA-STAD cohort. Tumor tissues exhibited overexpression of 17 genes (MCOLN1, MCOLN2, MCOLN3, PKD2L1, TRPC2, TRPC3, TRPM4, TRPM7, TRPM8, TRPV1, TRPV2, TRPV4, TRPV5, TRPC6, TRPC7, TRPM1, and TRPM2), while TRPV6 expression was higher in normal tissue (**Figure 2E**). High expression of TRPC1, TRPC3, TRPC4, TRPC6, TRPM3, MCOLN3, PKD2, TRPV2, TRPV4, and TRPV6 correlated with poorer overall survival (OS), whereas high TRPM4 expression predicted better outcomes (**Supplementary Figure 2**). Stage-specific analysis revealed differential expression of PKD1, PKD2, PKD2L1, TRPC1, TRPC3, TRPM7, and TRPV2, with peak levels in T4-stage tumors (**Supplementary Figure 3A**), implicating these genes in disease progression.

**Figure 3:**
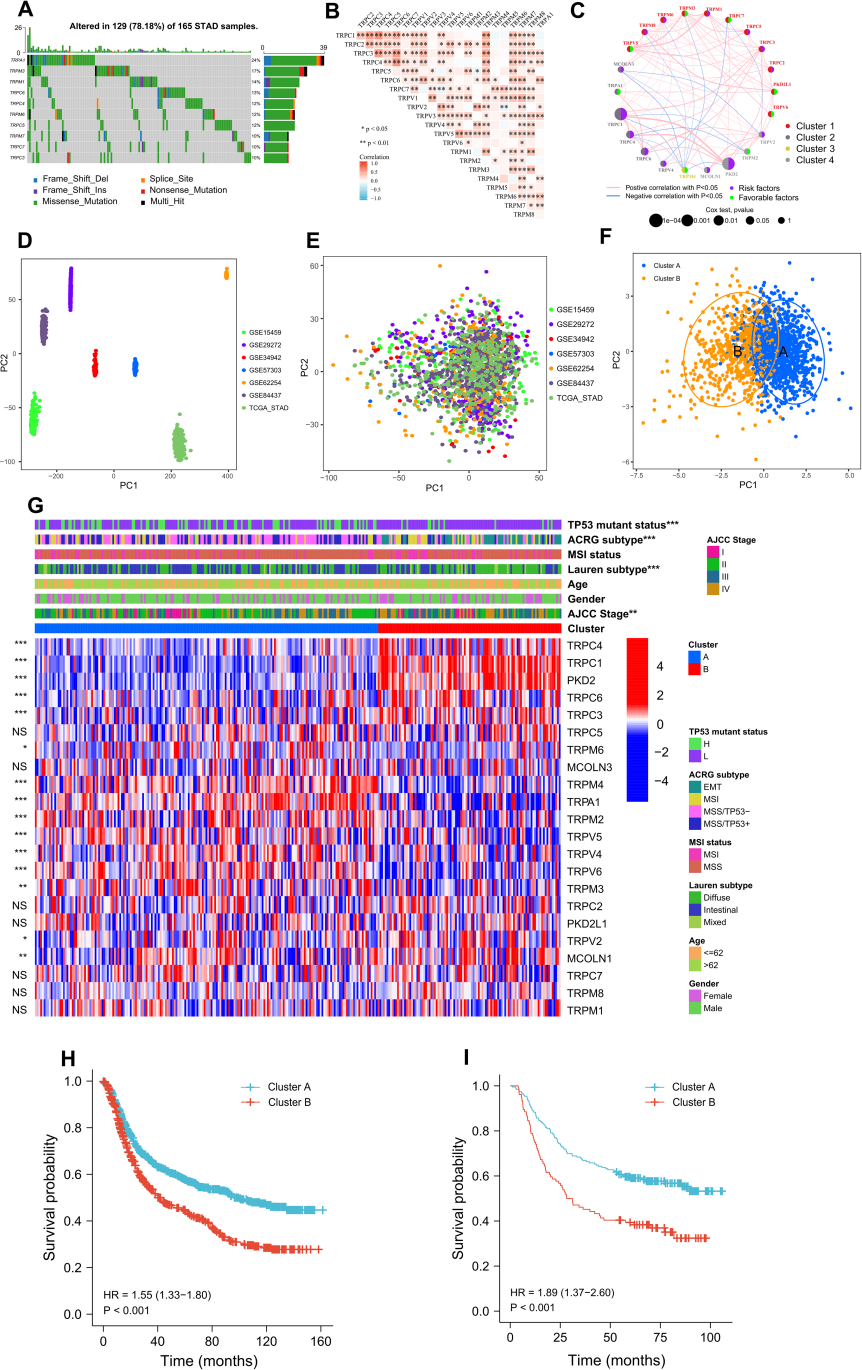
Predictive value of 22 TRP channel regulatory factors and TRP subtypes for clinical prognosis in GC patients. **(A)** The waterfall plot displaying the top 10 TRP channel regulators with the highest frequency of somatic mutations in the TCGA-STAD cohort. **(B)** Correlation analysis of the 22 TRP channel regulators within the TCGA-STAD cohort. **(C)** Based on Cox regression testing and correlation analysis across 1544 samples, the 22 TRP channel regulators were clustered into four groups, represented by different colors (**Supplementary Table 2**). **(D, E)** PCA illustrating the distribution of gene expression in seven GC cohorts (six GEO datasets and one from the TCGA database) before correction **(D)** and after correction **(E)**. **(F)** PCA revealing the relationships between two GC subtypes and the expression levels of the 22 TRP channel regulators. **(G)** Correlation analysis of two TRP subtypes with other GC subtypes, along with differences in the expression of the 22 TRP channel regulators in the GSE62254 cohort (**Supplementary Table 3**). **(H)** Comparison of OS between two TRP subtypes in the TCGA-STAD cohort. **(I)** Comparison of OS between two TRP subtypes in the GSE62254 cohort.

To elucidate molecular mechanisms, we integrated 6 GEO datasets with TCGA-STAD, analyzing 1544 samples encompassing 22 TRP regulators. Among 165 TCGA-STAD cases, 129 (78.18%) harbored TRP channel mutations, predominantly missense variants, with TRPA1 (24%), TRPM3 (17%), and TRPM1 (14%) most frequently altered (**Figure 3A**). Co-expression analysis demonstrated strong positive correlations, particularly between TRPC4–TRPC1/TRPC3 and TRPC6–TRPC3/TRPC4 (**Figure 3B**). Network-based assessment of 1544 samples identified TRPC1, TRPC4, TRPC6, and PKD2 as independent prognostic risk factors (**Figures 3C**, **Supplementary Table 2**). These findings establish TRP channels as cooperative mediators of GC.

We harmonized seven GC datasets using batch correction, with PCA confirming effective mitigation of technical variation (**Figures 3D, E**). Unsupervised clustering optimally stratified patients into two subtypes (Cluster A and B; **Supplementary Figures 3B–D**), validated by PCA and t-SNE visualization of TRP regulator expression patterns (**Figure 3F**, **Supplementary Figure 4A**). Cluster A demonstrated elevated expression of TRPM4, TRPA1, TRPM2, TRPV5, TRPV4, TRPV6, TRPM3, and TRPM6, while Cluster B overexpressed TRPV2, TRPC4, TRPC1, PKD2, TRPC6, TRPC3, and MCOLN1 (**Figures 3G**, **Supplementary Table 3**). Clinically, Cluster A was enriched for early-stage (AJCC I-II) tumors (72.5% vs 42.1% in Cluster B), intestinal-type histology (Lauren classification), and MSS/TP53^±^ molecular subtypes. Conversely, Cluster B predominated in advanced-stage (III-IV) disease (57.9%), diffuse/mixed histology, and EMT/MSI subtypes (**Supplementary Table 3**). Furthermore, survival analysis revealed significantly better outcomes for Cluster A in both TCGA-STAD (HR 0.48, 95% CI 0.32-0.71; P<0.001) and GSE62254 (HR 2.1, 95% CI 1.5-3.0; P<0.001) cohorts (**Figures 3H, I**). Collectively, these findings suggest TRP regulators may inform molecular subtyping and therapeutic strategies in GC.

### TRP channels shape TME in GC

**Figure 4:**
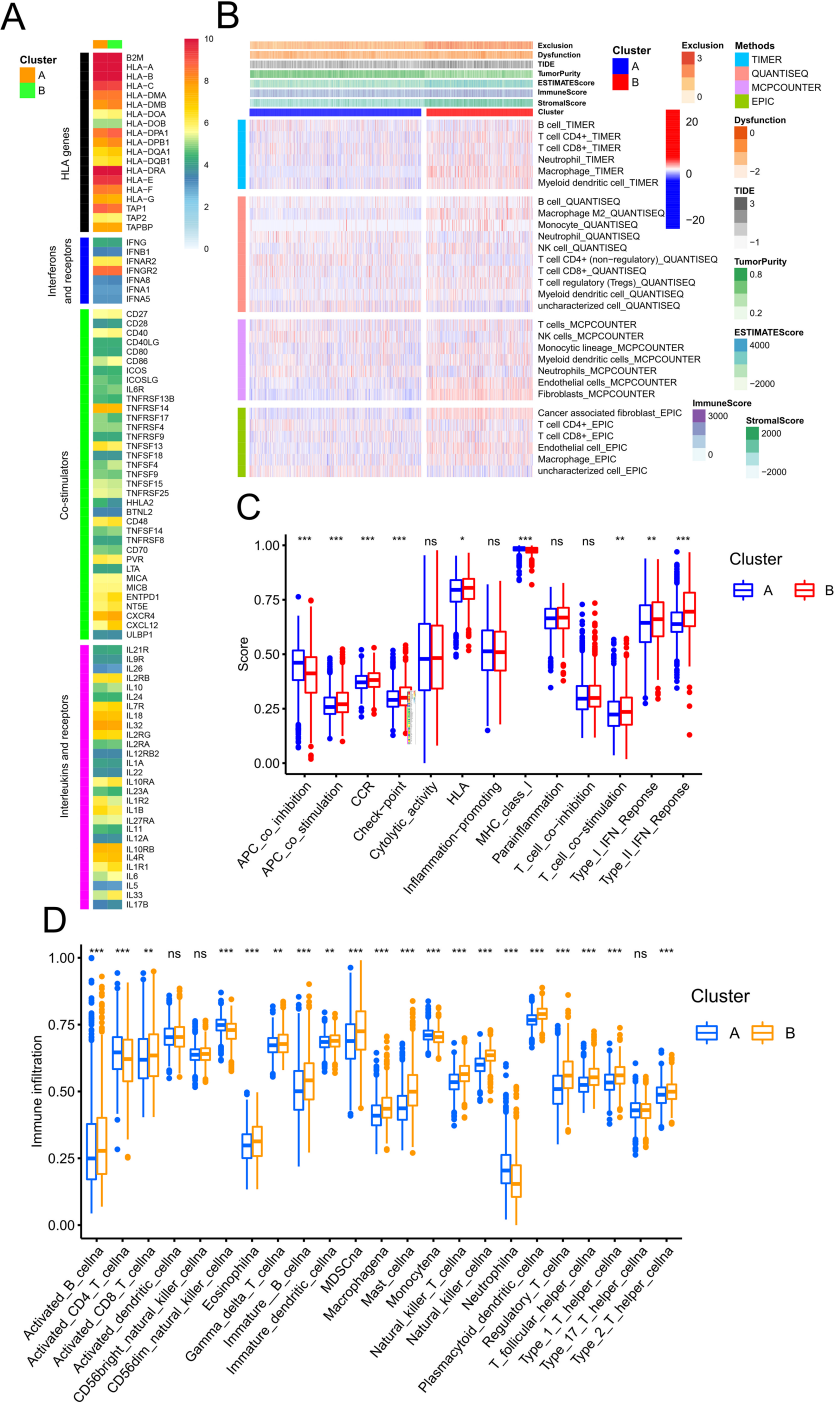
Correlation analysis between GC TRP subtypes and TME as well as tumor immune function. **(A)** The heatmap illustrating the mean expression levels of HLA genes, interferons, co-stimulators, and interleukins between two TRP subtypes. **(B)** Distribution of immune cell infiltration and immune scores between two TRP subtypes. **(C)** The boxplot depicting differences in key immune pathway scores between two TRP subtypes. **(D)** The boxplot showing differences in immune cell infiltration and immune function scores between the two TRP subtypes, as derived from ssGSEA analysis. **(C, D)** The central line represents the median value. The bottom and top of the boxes are the 25th and 75th percentiles (interquartile range). The whiskers encompass 1.5 times the interquartile range, The statistical significance was tested via Wilcoxon test. *p < 0.05; **p < 0.01; ***p < 0.001; ns P > 0.05.

In this study, we investigated the role of TRP channel regulators in shaping TME in GC. ClusterB exhibited elevated expression of HLA genes (B2M, HLA-DMB, HLA-DOA, HLA-DPA1, HLA-DPB1, HLA-DQA1, HLA-DQB1, HLA-DRA, HLA-E), while ClusterA showed higher expression of HLA-A, HLA-C, HLA-F, HLA-G, TAP1, TAP2, and TAPBP. Key immune function genes—including interferons and their receptors, co-stimulators, interleukins and receptors, co-inhibitors, chemokines, and chemokine receptors—were also more highly expressed in ClusterB (**Figure 4A**, **Supplementary Figures 4B**, **Supplementary Table 4**).

Using bull RNA-seq data, we assessed immune cell infiltration via TIMER, QUANTISEQ, MCPCOUNTER, EPIC, CIBERSORT, and XCELL. ClusterB demonstrated greater enrichment of CD8^+^ T cells, myeloid dendritic cells, tumor-associated fibroblasts, endothelial cells, and M2 macrophages, along with higher immune, stromal, dysfunction, and exclusion scores (**Figure 4B**, **Supplementary Figures 4C**, **Supplementary Table 5**). Additionally, ClusterB exhibited elevated activity in critical immune pathways, such as APC-co-stimulation, CCR, checkpoint, HLA, T-cell-co-stimulation, and type I/II IFN response pathways (**Figure 4C**). The ssGSEA further confirmed increased immune cell infiltration and function in ClusterB, except for activated_CD4_T_cellna, CD56dim-natural killer Cellna, Monocyte, and Neutrophilna (**Figure 4D**). These findings suggest that ClusterB has heightened immune cell activity, function, and pathway engagement compared to ClusterA. However, the presence of M2 macrophages, and regulatory T cells—along with elevated checkpoint pathway scores, stromal scores, and dysfunction/exclusion scores—implies potential immune escape or immunosuppression. Next, TRPV4, TRPV2, TRPC6, TRPC3, TRPC1, PKD2L1, PKD2, and MCOLN1 were significantly associated with most immune cell infiltration (**Supplementary Figure 4D**). Our results highlight the role of TRP channels in defining distinct TME in GC. These findings suggest complex role for TRP channels in modulating the GC immune microenvironment.

### TRP subtypes signaling differences and DEG analysis in GC

**Figure 5:**
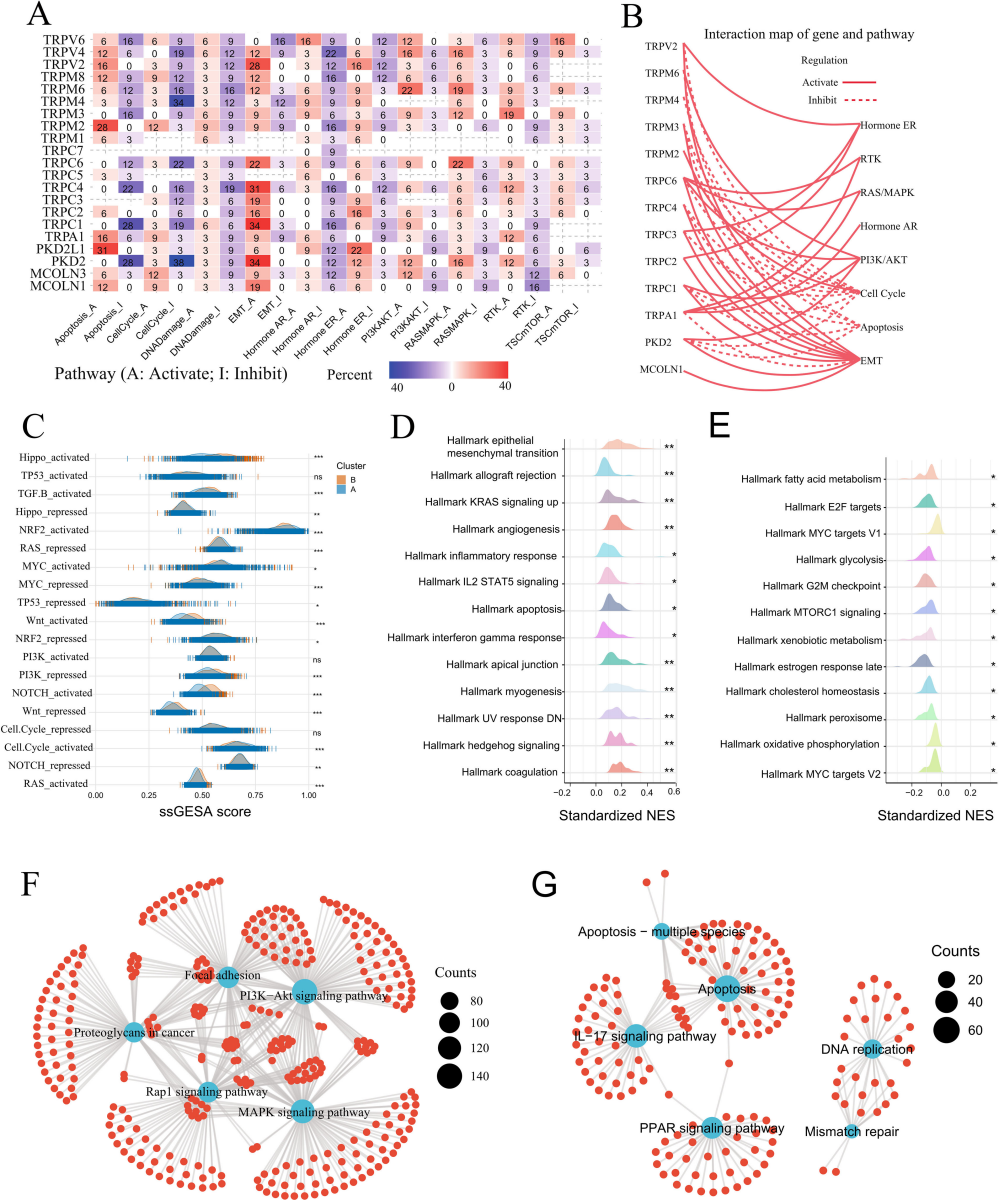
Functional analysis of TRP subtype in GC. **(A)** The correlation between the expression levels of the 22 TRP channel regulators and cancer signaling pathways was examined. The percentage indicates the proportion of cancers where TRP channel regulators influence pathway activity. Red represents pathway activation, while blue represents pathway inhibition. **(B)** The correlation between the 13 TRP channel regulators in GC and key cancer signaling pathways was analyzed. Solid lines indicate activation, while dashed lines indicate inhibition. **(C)** The ridge plot illustrates differences in GC characteristic pathway scores between the two TRP subtypes. **(D, E)**. In GSEA enrichment analysis, the activated pathways **(D)** and inhibited pathways **(E)** of cluster A relative to cluster B were identified. **(F, G)** KEGG enrichment analysis of upregulated genes **(F)** and downregulated genes **(G)** that are differentially expressed between cluster A and cluster B was performed.

We examined gene expression differences between both TRP subtypes and their associated signaling pathways. Across cancers, TRPM2 and PKD2L1 were highly activated in apoptosis, while TRPV2, TRPC6, TRPC4, TRPC1, and PKD2 activated EMT but suppressed cell cycle, DNA damage response, and hormonal AR pathways (**Figure 5A**). In GC, TRPV2, TRPM3, TRPC6, TRPC4, TRPC3, TRPC2, TRPC1, PKD2, and MCOLN1 activated EMT, whereas TRPM4, TRPM3, TRPC4, TRPC1, and PKD2 inhibited cell cycle and apoptosis (**Figure 5B**).

Comparison of 10 oncogenic pathways in GC TRP subtypes revealed higher activation scores for Hippo, TGF-β, Wnt, NOTCH, and RAS in Cluster B, suggesting enhanced proliferation and invasion (**Figure 5C**). Conversely, Cluster A exhibited greater inhibition of Hippo, RAS, TP53, NOTCH, and MYC pathways (**Figures 5C**, **Supplementary Table 6**). GSEA demonstrated significant activation of immune/inflammatory pathways (allograft rejection, inflammatory response, IL2-STAT5 signaling, interferon gamma response) in Cluster A, alongside suppression of cell cycle-related pathways (E2F targets, MYC targets, G2M checkpoint, mTORC1 signaling) (**Figures 5D, E**, **Supplementary Table 7**).

We performed WGCNA on 8,605 DEGs between Cluster A and B, identifying seven modules (**Supplementary Figure 5A**). The turquoise module showed the strongest survival correlation (r = −0.15, p = 1×10^-9^), suggesting its prognostic relevance in GC (**Supplementary Figure 5B**). The green (r = 0.47, p = 3.2×10^-8^) and yellow (r = 0.87, p = 1.2×10^71^) modules also correlated with gene significance, unlike gray module (**Supplementary Figure 5C**). KEGG/GO analysis revealed upregulation of tumor-progression pathways (focal adhesion, PI3K-Akt, proteoglycans in cancer, Rap1, MAPK) and immune suppression, while downregulated genes were enriched in apoptosis, IL-17, DNA replication, PPAR signaling, and cell cycle inhibition (**Figures 5F, G**; **Supplementary Figures 5D, E**; **Supplementary Tables 8–S9**). In TCGA-STAD, Cluster A (n = 229, 91.7% mutation frequency) and Cluster B (n = 133, 81.95%) exhibited frequent TTN, TP53, and MUC16 missense mutations (**Supplementary Figure 5F**). Cluster B had higher somatic mutation frequency and TMB, potentially linked to DNA damage checkpoint dysregulation (**Supplementary Figure 5G**).

### Construction of TRPscore as a prognostic biomarker and TME modulator in GC

**Figure 6:**
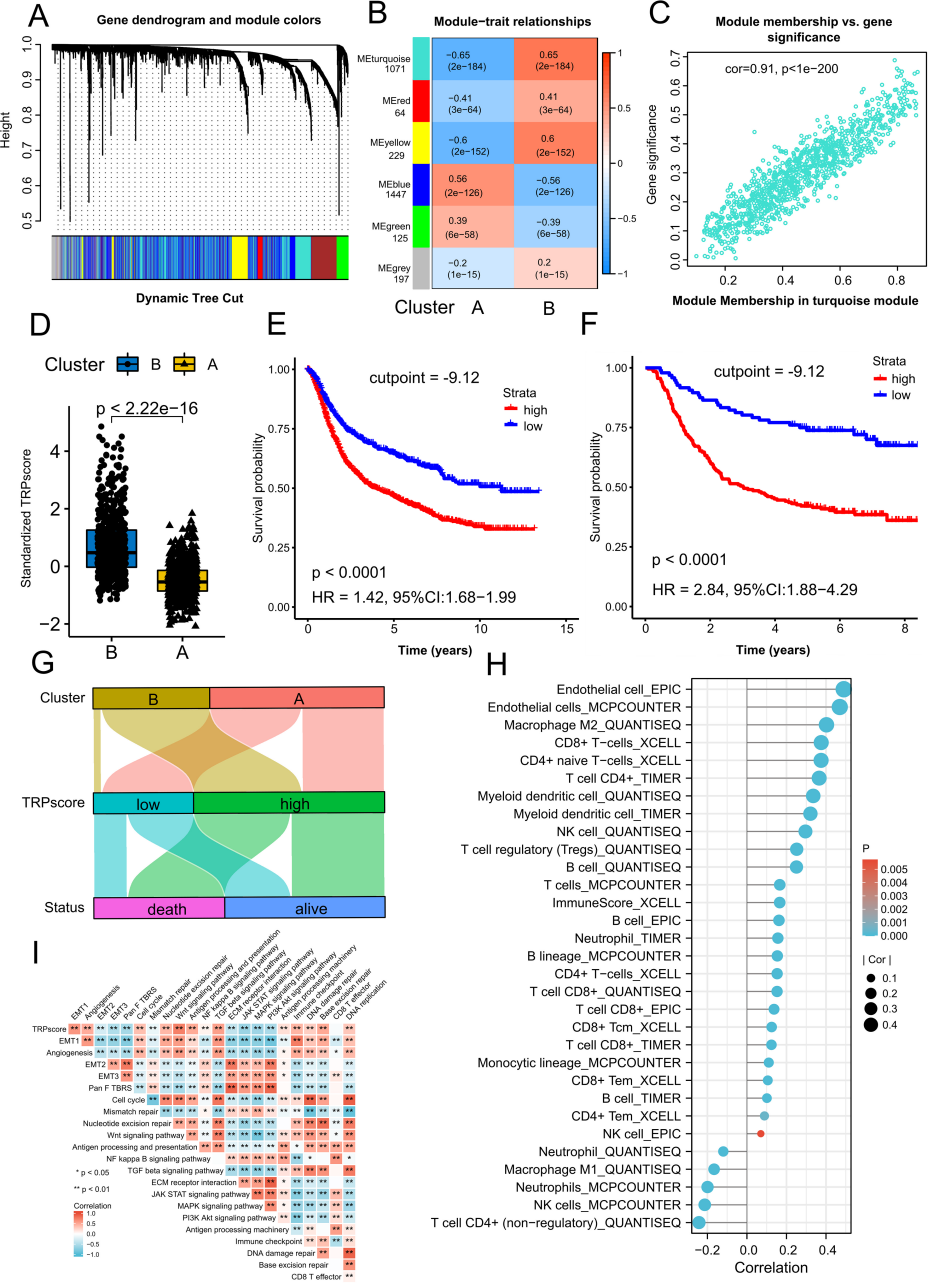
Construction of WGCNA and TRPscore for GC. **(A)** The cluster dendrogram was generated based on clustering analysis of 3172 survival-related genes. Each branch in the dendrogram represents a gene, and different colors indicate modules containing co-expressed weighted genes. **(B)** Heatmap illustrating the correlation between module characteristic genes and TRP subtypes in GC, with each column displaying the respective correlation coefficient and p-value. **(C)** Correlation between eigengenes of the turquoise module (n = 1071) and gene significance. **(D)** Comparison of TRPscore between Cluster A and Cluster B **(E, F)** Kaplan-Meier survival curves for high and low TRPscore groups in 1544 GC samples **(E)** and the GSE62254 cohort **(F)**. The optimal cutoff value for high and low TRPscore groups was determined using the survminer package in R **(G)** The Sankey diagram visualizes the relationship between TRP subtype, TRPscore, and survival. **(H)** The bubble plot shows the correlation between TRPscore and immune cell infiltration. Bubble color represents the P-value, and bubble size represents the strength of the correlation, with larger bubbles indicating stronger correlations. Immune cell infiltration was assessed using EPIC, MCPCOUNTER, QUANTISEQ, XCELL, and TIMER. **(I)** The Spearman rank correlation test was used to assess the correlation between TRPscore and scores of other key pathways. * p < 0.05; ** p < 0.01; *** p < 0.001; ns p > 0.05.

These results confirm the critical role of TRP channel regulators in the GC immune microenvironment and patient survival. We identified 3172 survival-associated DEGs for WGCNA, which clustered into five modules (**Figure 6A**). The turquoise module showed significant correlations with Cluster A (r = −0.65, p=2e-184) and Cluster B (r = 0.65, p=2e-184; **Figure 6B**). A strong correlation between turquoise module genes and gene significance was observed (r = 0.91, P < 1e–200; **Figure 6C**). To substantiate the functional coherence of the turquoise module with TRP channel biology, we performed GO biological process enrichment analysis on the 1,071 constituent genes. This revealed robust enrichment in canonical TRP-associated pathways, including calcium ion transport, calcium-mediated signaling, and cellular response to mechanical stimulus (**Supplementary Figures 6E**, **Supplementary Table 8**). Notably, the gene set exhibited significant enrichment for cell-substrate adhesion and positive regulation of PI3K-Akt signaling, processes mechanistically implicated in TRP-mediated oncogenic mechanisms and experimentally validated in our functional studies.

We using the PCA (orthogonal rotation) method to construct the TRPscore—a prognostic metric based on 1071 turquoise module genes. Cluster B had higher TRPscores than Cluster A (**Figure 6D**). Optimal stratification (survminer R package) divided 1544 patients into high- and low-TRPscore groups, with the latter showing superior survival (HR = 1.42, 95% CI: 1.18–1.99; P < 0.0001; **Figure 6E**). This prognostic value was validated in GSE62254 (HR = 1.68, 95% CI: 1.42–1.99; P < 0.0001; **Figure 6F**). Cluster B predominantly comprised high-TRPscore cases with higher mortality (**Figure 6G**).

TRPscore positively correlated with endothelial cell and M2 macrophage infiltration but inversely associated with neutrophils, M1 macrophages, NK cells, and non-regulatory CD4^+^ T cells (**Figure 6H**). Pathway correlation analyses identified significant positive relationships of TRPscore with key proliferative/immune pathways including cell cycle, Wnt signaling, antigen presentation, DNA repair pathways (**Figure 6I**). Furthermore, TRPscore negatively correlated with PKD2L1, TRPC7, TRPV5/6, TRPM2/4, and TRPA1, but positively with TRPC1/3/4/6, TRPV2, PKD2, MCOLN1/3, and TRPM6 (**Supplementary Figure 6A**). In GSE62254, low TRPscore correlated with early-stage (AJCC I–II), microsatellite instability-high (MSI-H) status, and improved survival, while high TRPscore associated with advanced disease (AJCC III–IV), mixed/EMT subtypes (Lauren/ACRG classifications), and worse outcomes (**Supplementary Figure 6B**). In addition, TRPscore was strongly correlated with most immune checkpoint-related genes (**Supplementary Figure 6C**). Low TRPscore samples overexpressed inhibitory checkpoints (SIGLEC15, PVR, LGALS9, CEACAM1), whereas high TRPscore correlated with activators (PDCD1LG2, CD33, TLR4, LY96; **Supplementary Figures 6C, D**), highlighting the role of TRPscore in immune regulation. These findings highlight TRPscore a robust prognostic biomarker reflective of immune microenvironment alterations and clinical outcomes in GC.

### TRPscore predicts the response of GC patients to immunotherapy

**Figure 7:**
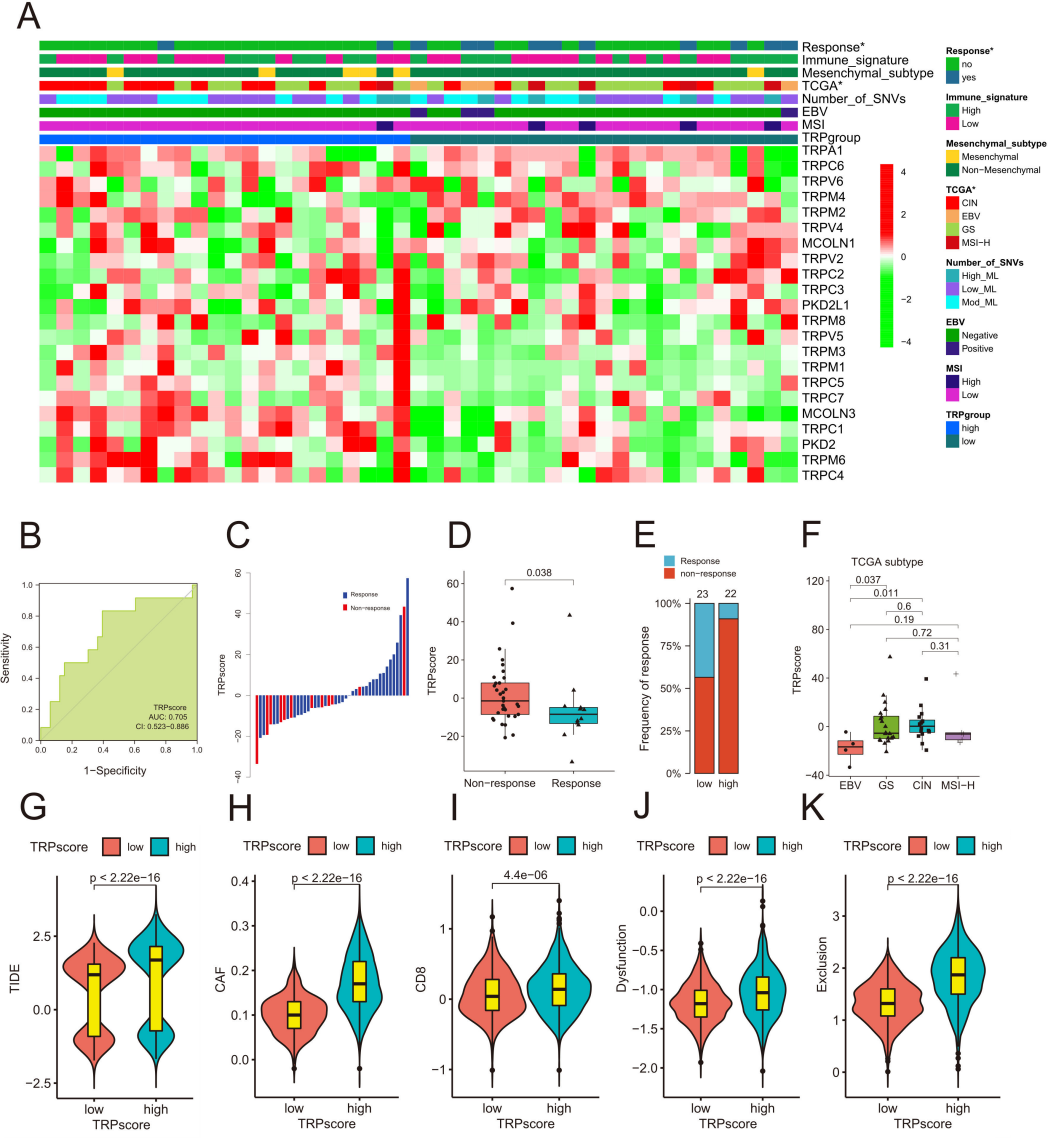
Evaluation of immune therapy response based on TRPscore. **(A)** The heatmap displays the relationship between TRPscore and the response to PD-1 blockade therapy, as well as GC subtypes, in the Kim cohort (**Supplementary Table 11**). **(B)** ROC curve analysis assessing the predictive value of TRPscore for patients undergoing PD-1 blockade therapy. **(C)** The waterfall plot illustrates the sequential relationship between TRPscore and the response to PD-1 blockade therapy. **(D)** Comparison of TRPscore between responders and non-responders in PD-1 blockade therapy. **(E)** Composition of responders in PD-1 blockade therapy among high-TRPscore and low-TRPscore groups. **(F)** Distribution of TRPscore across TCGA subtypes in GC. **(G–K)** Comparison of TIDE, CAF, CD8, Dysfunction, and Exclusion scores between high-TRPscore and low-TRPscore groups in 1544 samples.

To evaluate the role of TRPscore in predicting response to anti–PD-1 immunotherapy, we analyzed the Kim cohort using a combined heatmap to compare TRP channel regulator expression, TCGA molecular subtypes (31), and treatment response between high- and low-TRPscore groups (**Figure 7A**). The high-TRPscore group showed increased expression of TRPC5, TRPM1, TRPM3, TRPM6, MCOLN3, and TRPC1, whereas TRPM4 was downregulated (**Figure 7A**; **Supplementary Table 11**). The chromosomal instability (CIN) TCGA subtype was predominant in the high-TRPscore group, while the Epstein-Barr virus (EBV)–associated subtype was enriched in the low-TRPscore group. TRPscore demonstrated moderate predictive performance for anti–PD-1 response (area under the curve [AUC], 0.705; **Figures 7B, C**), with low-TRPscore patients exhibiting greater therapeutic benefit. Responders to PD-1 blockade were more frequently observed in the low-TRPscore group (**Figures 7A, C, D**). To address the limitation of restricted GC immunotherapy datasets, we expanded our study to multiple independent cohorts covering melanoma, kidney, and non-small cell lung cancers (31). TRPscore similarly predicted treatment response across these various cancers (**Supplementary Figure 7D**), indicating that its relationship with ICB sensitivity is a biologically conserved feature of the TME. This confirmation indirectly supports the credibility of our gastric cancer findings and motivated us for searching into the molecular basis of TRP-mediated immunomodulation. These findings were further validated by lower TRPscore levels in responders to PD-1 therapy, EBV-positive tumors, and non-mesenchymal samples (**Figures 7D–F**; **Supplementary Figure 7A**).

Evaluation of TIDE-related markers in 1544 gastric samples revealed higher TIDE, CAF, CD8, Dysfunction, Exclusion, Merck18, and TAM-M2 expression in high-TRPscore cases, all positively correlated with TRPscore. Conversely, MSI-Expr-Sig and MDSCs were inversely associated (**Figures 7G–K**, **Supplementary Figures 7B, C**). TRPscore is associated with distinct TME features and can serve as a moderate predictor of diminished response to anti–PD-1 immunotherapy in GC.

### Functional experiments and single-cell expression profiling of TRPV2 in GC

Our analysis demonstrated significantly elevated TRPV2 expression in tumor tissues than normal tissues (**Figure 2E**), predicted poor prognosis (**Supplementary Figure 2**), and peaked in T4-stage tumors (**Supplementary Figure 3A**), suggesting a role in disease progression. Mechanistically, TRPV2 activation correlated with PI3K/AKT signaling (**Figure 5B**)—a key pathway driving proliferation, metastasis, and therapy resistance (32–34)—and pan-cancer analysis revealed strong association between TRPV2 expression and EMT pathway activation (**Figure 5A**). These findings suggest that TRPV2 may play a critical role in GC development and progression.

**Figure 8:**
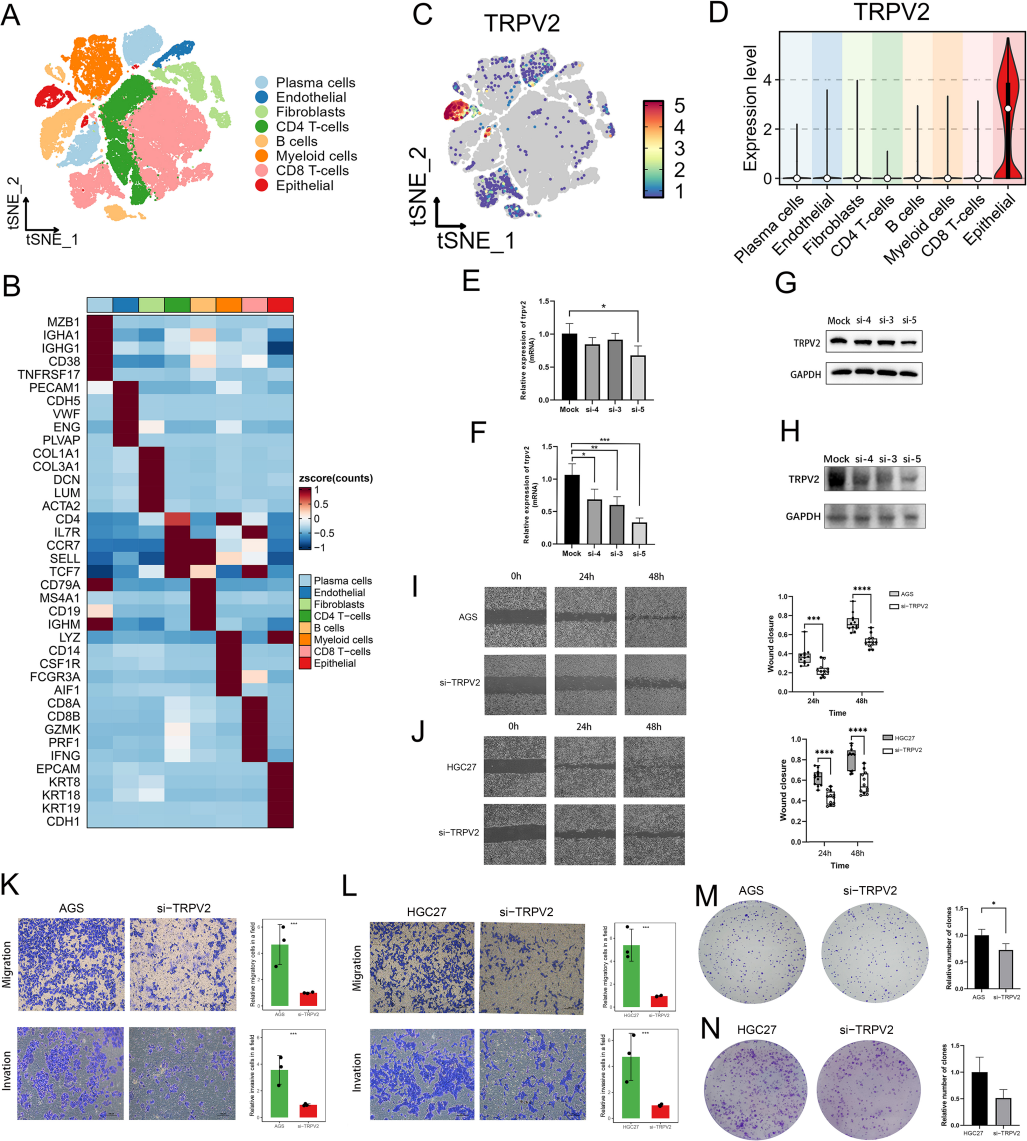
scRNA-seq analysis and functional characterization of TRPV2 in GC. **(A)** t-distributed stochastic neighbor embedding (tSNE) projection of 59,594 single cells from 21 primary GC samples (GSE206785). **(B)** Heatmap showing gene expression patterns of cell-type marker genes in scRNA-seq data. **(C)** tSNE plot displaying spatial distribution of TRPV2 expression across cell populations. **(D)** Violin plots showing TRPV2 expression levels in eight major cell types. **(E, F)**. Quantitative RT-PCR analysis of TRPV2 mRNA expression levels in AGS **(E)** and HGC-27 **(F)** cell lines. **(G, H)**. Western blot analysis of TRPV2 protein levels in AGS **(G)** and HGC-27 **(H)** cell lines. **(I–J)**. Scratch assay showing reduced migration ability of AGS **(I)** and HGC-27 **(J)** cells after TRPV2 downregulation with quantitative analysis (right panels). **(K, L)**. Transwell assay demonstrating suppressed migration and invasion abilities of AGS **(K)** and HGC-27 **(L)** cells after TRPV2 downregulation. M-N. Colony formation assay indicating reduced colony formation ability of AGS **(M)** and HGC-27 **(N)** cells after TRPV2 downregulation. * p < 0.05; **p< 0.01; *** p < 0.001; **** p < 0.0001.

To determine whether TRPV2 was primarily expressed in epithelial cells within tumor tissues, we analyzed scRNA-seq data (GSE206785) (17). As reported in our previous work (32), TRPV2 was specifically enriched in epithelial cells (**Figures 8A–D**). To explore the functional role of TRPV2 in GC, we performed *in vitro* experiments. TRPV2 was effectively knocked down in two GC cell lines, AGS and HGC27, using siRNA (**Figures 8E–H**), with si-5 selected for further studies. Knockdown of TRPV2 significantly reduced wound healing capacity in scratch assays (**Figures 8I, J**), impaired migration and invasion in transwell assays (**Figures 8K, L**), and suppressed clonogenic ability in colony formation assays (**Figures 8M, N**). These results demonstrate that TRPV2 promotes malignant behaviors in GC cells, including migration, invasion, and proliferation.

### Establishment of nomogram for predicting survival

**Figure 9:**
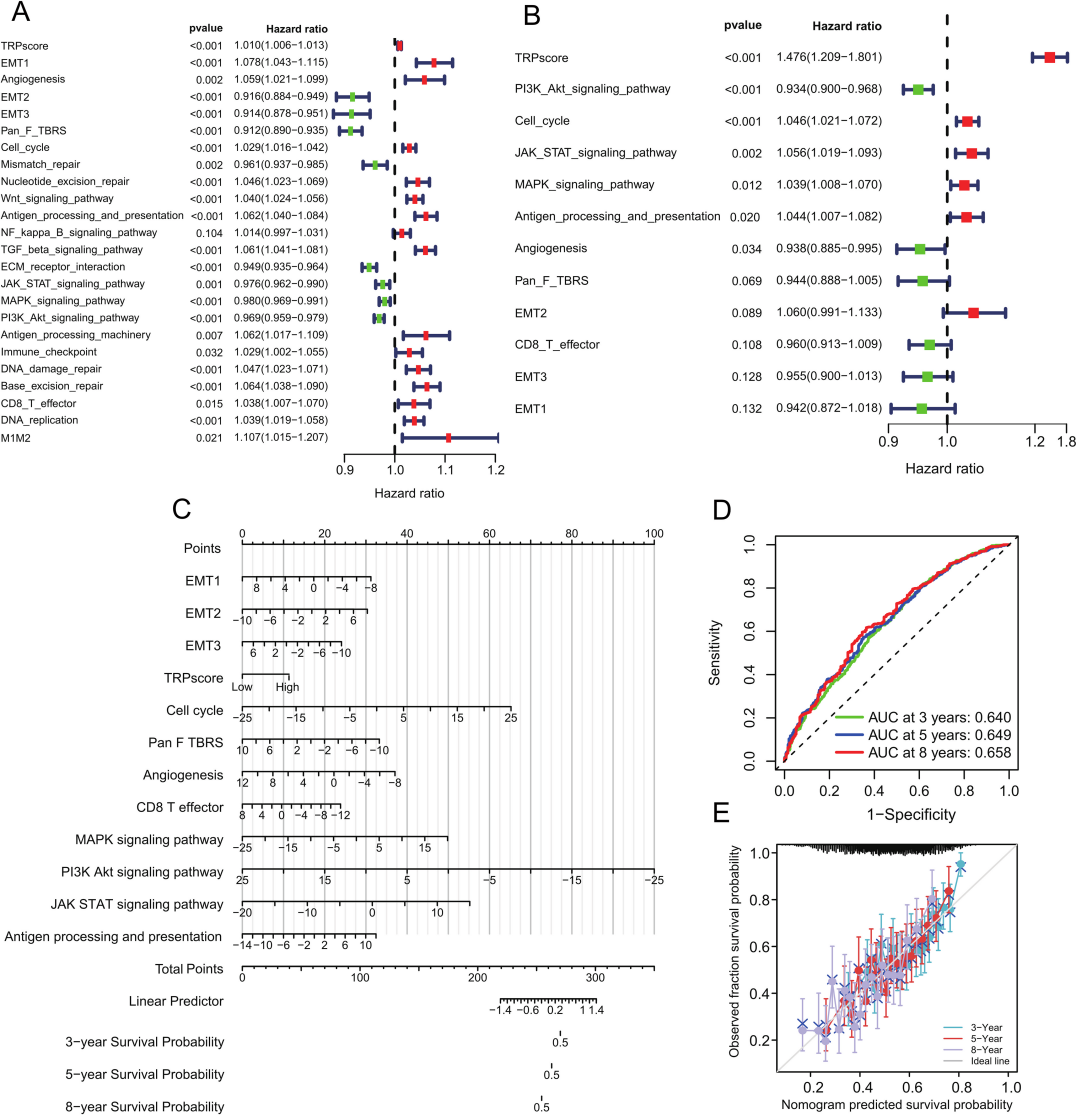
The impact of TRPscore and key signaling pathways on GC occurrence and survival. **(A, B)** Univariate and multivariate Cox regression analyses. A hazard ratio (HR) > 1 indicates risk factors, while HR < 1 indicates protective factors. **(C)** Nomogram integrating the results of Cox regression analysis. **(D)** ROC curves for the nomogram predicting 3-, 5-, and 8-year survival probabilities. **(E)** Calibration curves for the nomogram predicting 3-, 5-, and 8-year survival probabilities. The y-axis represents the measured survival probabilities, while the x-axis represents the nomogram-predicted survival probabilities. The diagonal dotted line represents perfect prediction by an ideal model, and a closer fit to this line indicates better predictive performance.

Univariate Cox regression analysis identified TRPscore and multiple signaling pathways as significant predictors of poor survival in GC (**Figure 9A**). Multivariate analysis confirmed TRPscore as an independent prognostic factor (HR = 1.476, 95% CI: 1.209-1.801; p<0.001; **Figure 9B**). Using these results, we developed a comprehensive nomogram incorporating twelve independent variables: EMT1-3, TRPscore, cell cycle, Pan-F-TBRs, angiogenesis, CD8^+^ T effector status, and MAPK, PI3K-Akt, JAK-STAT signaling pathways, along with antigen processing and presentation (**Figure 9C**). The nomogram demonstrated reliable predictive performance, with ROC analysis showing AUC values of 0.640 (3-year), 0.649 (5-year), and 0.658 (8-year survival) (**Figure 9D**). Calibration plots confirmed excellent agreement between predicted and observed outcomes (**Figure 9E**). This tool provides clinicians with a quantitative method to assess individual patient prognosis and guide clinical decision-making.

## Discussion

Previous studies have established a significant association between TRP pathway modulators and the TME, suggesting their potential as biomarkers for tumor immunotherapy (33). However, the mechanisms by which these modulators influence the TME in GC remain poorly defined. Characterizing TRP immune phenotypes and TRP scores within the GC TME may therefore elucidate how TRP channels affect antitumor immunity and could inform strategies to enhance current immunotherapies.

Growing evidence underscores the importance of TRP channel modulators in cancer (34). In this study, we evaluated the genetic and transcriptional profiles of 28 TRP channel modulators across normal and tumor tissues from 33 cancer types, revealing dysregulated expression patterns potentially linked to genomic variation. These findings align with a previous study that comprehensively mapped genomic and transcriptomic alterations of TRP channels across 33 cancers, providing a valuable resource for mechanistic and therapeutic investigation (35). Identifying TRP genes with frequent genetic alterations may help advance predictive, and personalized therapeutic strategies in oncology.

Based on data from 1544 GC patients, we identified two distinct TRP subtypes. Cluster A was associated with a superior survival outcome. Comparative analyses revealed substantial differences in immune cell infiltration and pathway activity between subtypes. Although Cluster B exhibited higher immune cell infiltration, immune function scores, and immune pathway activation, it also displayed elevated stromal scores and increased abundance of fibroblasts, endothelial cells, regulatory T cells, and M2 macrophages. Pathway analysis further indicated marked activation of the Notch and Hippo signaling pathways in Cluster B, which have been implicated in promoting proliferation, invasion, and immune modulation in GC (36, 37). In contrast, genes upregulated in Cluster A were enriched in focal adhesion, PI3K-Akt signaling, and negative regulation of immune effector processes (38). These findings suggest a potential link between Cluster A and responsiveness to immunotherapy. Focal adhesion kinase acts upstream of PI3K-Akt signaling, a pathway frequently altered in cancers (39). Its inhibition may suppress tumor progression, while blocking negative immune regulatory mechanisms could potentiate immunotherapy efficacy.

Given the critical role of TRP channel regulators in GC immunity and the heterogeneity among TRP subtypes, it is essential to characterize their expression patterns in patients. We developed a TRPscore based on turquoise module genes significantly associated with survival. Patients with low TRPscores exhibited better prognosis than those with high TRPscores, and most Cluster B cases fell into the high-TRPscore group. TRPscore showed promise as a predictor of response to PD-1 blockade therapy, with a higher proportion of responders in the low-TRPscore group. Compared to TCGA subtypes, the EBV subtype was predominantly associated with low TRPscores. EBV-positive patients, who demonstrated a 100% objective response rate to PD-1 blockade (40), had significantly lower TRPscores.

Notably, LY96—an immune-related gene—was positively correlated with TRPscore and highly expressed in the high-TRPscore group (**Supplementary Figure 6C**). Previous studies reported a negative correlation between LY96 and both MSI and TMB in STAD, and a positive correlation with the IC_50_ of certain chemotherapeutic agents, suggesting that high LY96 expression may confer treatment resistance (41). We thus hypothesize that elevated TRPscore may correspond to increased LY96 expression and reduced immunotherapy responsiveness. Consistent with this, the high-TRPscore group exhibited elevated TIDE, CAF, Dysfunction, Exclusion, TAM-M2, and Merck18 scores, but lower MSI scores—all indicative of diminished immune response. Interestingly, this group also had higher CD8 scores and lower MDSC scores. The differential response to immunotherapy may stem from more active immune evasion in high-TRPscore tumors versus a less immunosuppressive microenvironment in low-TRPscore cases.

In the TCGA-STAD cohort, TRPC1, TRPC3, and TRPC6 expression levels were significantly positively correlated with one another. Elevated expression of these genes was associated with worse prognosis. Furthermore, these genes were highly expressed in Cluster B and showed a significant positive correlation with TRPscore. These results suggest a potential synergistic adverse effect of TRPC1, TRPC3, and TRPC6 in GC, consistent with the report by Ge et al (10). Similarly, TRPV4 expression was also associated with tumor progression, corroborating findings by Wang et al., in which suppression of TRPV4 promoted apoptosis and inhibited migration in GC cells (42).

TRPV2 and TRPM4 showed opposing associations with survival outcomes, pathway activation, TRP subtype relationships, and TRPscore. In cellular experiments, inhibiting TRPV2 in GC cell lines AGS and HGC27 reduced their migration and invasion capabilities. This finding is consistent with studies on the role of TRPV2 in esophageal squamous cell carcinoma, liver cancer, and GC (43–45). Similarly, TRPM4 has been studied in prostate, colorectal, cervical, and breast cancers (46, 47). However, the mechanisms underlying the roles of TRPV2 and TRPM4 in GC remain poorly understood. Therefore, further investigations are needed to elucidate these roles and mechanisms in GC.

The TRP-related molecular subtypes and quantitative TRPscore found in this work have potential for precision oncology in gastric cancer (GC). First, the TRP-based classification adds a new dimension to clinicopathological frameworks including ACRG molecular subtypes, which have shown prognostic superiority over conventional histology (15) by identifying aggressive disease subsets like Cluster B that may be missed by staging alone. Second, TRPscore independently predicts survival outcomes while quantifying immune microenvironment characteristics and potential responsiveness to immune checkpoint blockade, enabling individualized therapeutic stratification analogous to molecular profiling for PD-1 inhibitor prediction (40). Based on these principles, clinical translation should follow two strategies. Following the successful paradigm of biomarker-enriched trial designs (48), prospective cohorts receiving anti-PD-1 immunotherapy should validate TRPscore (particularly low scores) as a predictive biomarker for treatment efficacy and explore its combinatorial utility with established markers like PD-L1 and MSI. Functionally validated targets like TRPV2 provide a mechanistic rationale and patient selection framework for biomarker-enriched trials of novel TRP channel antagonists targeting Cluster B-enriched patient populations, as shown by TRPV6-targeted therapy (49).

While we delineated TRP-expression landscapes and their clinical weight, their upstream governors remain putative: TRPV2 promoter methylation anticorrelates with its transcript level, consistent with reports that DNA methylation mediates TRP channel dysregulation in gastrointestinal malignancies (30) and Cluster B’s EMT signature points to a common transcriptional master switch (50). Future research should advance along two directions. On the mechanistic front, translating these correlative findings into causal insights will require epigenetic editing, chromatin analysis, and cell-cell interaction models. On the translational front, developing clinical-grade TRP signature assays and validating the utility of TRP-based subtyping in prospective clinical trials are essential steps toward precision oncology implementation.

This study has several limitations. First, all population data were derived from public databases; prospective real-world data are needed to further validate the clinical relevance of our findings. Second, although we verified the role of TRPV2 at the cellular level, additional *in vitro* and *in vivo* experiments are required to elucidate the molecular mechanisms by which various TRP channels influence the development and progression of GC. Third, despite the AUC values not exceeding 0.7 (0.640, 0.649, and 0.658 for 3-, 5-, and 8-year survival, respectively), the model demonstrates clinically relevant predictive ability. In the context of highly heterogeneous malignancies like GC, an AUC above 0.6 is considered to provide moderate yet meaningful discriminatory power (51–53). The principal strength of our nomogram lies in its integration of the TRPscore with key pathway activities, offering a multidimensional assessment of tumor biology. Moreover, the excellent calibration observed (**Figure 9E**) underscores its reliability in estimating individual survival probabilities. We acknowledge the model’s limitations: most datasets included confounding variables such as radiotherapy, chemotherapy, neoadjuvant therapy, and surgery, which may have influenced the assessment of immune responses and the prognostic relevance of TRP-related molecules. To address these constraints, we plan to incorporate additional data dimensions and conduct prospective validation in future studies.

## Conclusions

This study identified two TRP-related molecular subtypes in GC with distinct survival outcomes, immune infiltration, and pathway activation. A novel TRPscore was developed to quantify TRP regulator expression and immune infiltration in patients with GC. This score helped define immune phenotypes, predict prognosis, and estimate potential response to immunotherapy. ScRNA-seq revealed that TRPV2 was primarily overexpressed in epithelial cells and associated with PI3K/AKT signaling and epithelial-mesenchymal transition. *In vitro* experiments showed that TRPV2 knockdown suppressed migration, invasion, and clonogenicity. Incorporating TRPscore and key pathway signatures into a nomogram enabled accurate individualized survival prediction.

## Data Availability

The original contributions presented in the study are included in the article/**Supplementary Material**. Further inquiries can be directed to the corresponding authors.

## Supplementary material

Supplementary Figure 1 Genomic alterations and survival associations of TRP channel regulators across cancer types. **(A)** Bubble chart showing the differences in methylation levels of 28 TRP channel regulators between tumor and normal tissues across cancers. Methylation Diff (T−N): the mean methylation level of tumor samples minus that of normal samples. **(B)** The heterozygous CNV bubble chart displays the percentages of heterozygous CNV amplifications and deletions for each gene across cancers. **(C)** The heterozygous CNV bubble chart illustrates the percentages of homozygous CNV amplifications and deletions for each gene across cancers. **(D)** The location of CNV alterations for the 28 TRP channel regulators on the 23 chromosomes in the STAD cohort. **(E)** CNV pie charts depict the distribution of CNV categories (heterozygous and homozygous amplification/deletion) for each gene in selected cancers. Each pie represents the proportion of CNV types for TRP channel regulators in a specific cancer type, with different colors corresponding to distinct CNV types. None: no CNV detected. **(F)** Bubble chart presenting survival differences between patients with high and low expression of TRP channel regulators across cancers. Survival analysis was performed using the Cox proportional hazards model to calculate HRs for each gene, followed by Kaplan–Meier survival analysis with a log-rank test. Bubbles on the solid line indicating statistical significance (P ≤ 0.05).

Supplementary Figure 2 The survival differences between the high- and low-expression groups of the 28 TRP channel regulators were determined using the maximum Youden index as the cut-off value.

Supplementary Figure 3 Integration of consensus distribution analysis and heatmap visualization supports a two-cluster classification for GC patients based on TRP regulator expression. **(A)** Boxplot analysis of 28 TRP channel regulators across T stages in the STAD cohort was shown. **(B)** The relative change in area under the cumulative distribution function (CDF) curve was calculated by comparing cluster numbers K and K−1. For K = 2, the total area under the curve was shown. The optimal K was selected based on the relative increase in consensus. **(C)** Colored lines represented the CDF of the consensus matrix for each K, derived from a 100-bin histogram. The CDF plateau indicated maximal consensus and cluster stability. **(D)** Consensus matrix heatmaps for K = 2 to K = 7 were generated using 22 TRP regulators. Blue indicated consistent co-clustering, white indicates consistent separation, and gradations reflect clustering ambiguity. The optimal number of clusters for GC patients was identified as two.

Supplementary Figure 4 TRP subtypes and immune landscape in GC. **(A)** The tSNE plot demonstrates that the two TRP subtypes are characterized by distinct expression levels of the 22 TRP channel regulators. **(B)** Comparison of key tumor immune function genes between two TRP subtypes. **(C)** Distribution of immune cell infiltration and immune scores between two TRP subtypes. **(D)** Correlation analysis of immune cell infiltration and immune function scores with the 22 TRP channel regulators based on ssGSEA analysis. The correlation analysis was conducted using the two-tailed Spearman correlation.

Supplementary Figure 5 Comprehensive molecular characterization and survival analysis of GC clusters. **(A)** The cluster dendrogram was generated based on the differential expression of 8605 genes between Cluster A and Cluster B. Each branch in the dendrogram represents a gene, and different colors indicate modules containing co-expressed weighted genes. **(B)** Heatmap illustrating the correlation between module characteristic genes and survival in GC, with each column displaying the respective correlation coefficient and p-value. Among these, a total of 3172 genes were found to be significantly associated with survival (p < 0.05), including genes in the turquoise module (2010), yellow module (328), blue module (676), and black module (158). **(C)** Correlation analysis between characteristic genes of the green module, grey module, red module, and yellow module and gene significance. **(D, E)** GO enrichment analysis was performed for genes upregulated in Cluster A compared to Cluster B **(D)** and genes downregulated in Cluster A compared to Cluster B **(E, F)** The distribution of tumor mutation burden frequency and types in Cluster A and Cluster B within the TCGA-STAD dataset. **(G)** Comparison of tumor mutation burden (TMB) between Cluster A and Cluster B.

Supplementary Figure 6 TRPscore and its association with immune checkpoint expression in GC. **(A)** Correlation between TRPscore and 22 TRP channel regulatory factors. **(B)** Relationship between TRPscore and other subtypes of GC as well as clinical characteristics in the GSE62254 cohort. **(C)** Correlation between TRPscore and immune checkpoint-associated genes. **(D)** Differential comparison of immune checkpoint-associated genes between high-TRPscore and low-TRPscore groups. **(E)** GO biological process (BP) enrichment of the 1,071 turquoise module genes (TRPscore-related), showing significant association with calcium ion transport, calcium-mediated signaling, and PI3K-Akt signal transduction (adjusted P < 0.05).

Supplementary Figure 7 Evaluation of immune therapy response based on TRPscore. **(A)** Distribution of TRPscore across EBV, Immune Signature, Mesenchymal, MSI, and Number of SNVs in GC. **(B)** Comparison of MDSC, Merck18, MSI, TAM_M2, and IFNG between high-TRPscore and low-TRPscore groups in 1544 samples. **(C)** Correlation analysis of TRPscore with TIDE-related predictive indicators. **(D)** Cross-cancer validation of TRPscore for predicting immune checkpoint blockade response. Receiver operating characteristic (ROC) curves for three independent cohorts: renal cell carcinoma, melanoma, and non-small cell lung cancer.

## References

[1] Eom SS Ryu KW Han HS Kong SH . A comprehensive and comparative review of global gastric cancer treatment guidelines: 2024 update. J Gastric Cancer. (2025) 25:153–76. doi: 10.5230/jgc.2025.25.e10, PMID: 39822173 PMC11739642

[2] Sung H Ferlay J Siegel RL Laversanne M Soerjomataram I Jemal A . Global cancer statistics 2020: GLOBOCAN estimates of incidence and mortality worldwide for 36 cancers in 185 countries. Ca-Cancer J Clin. (2021) 71:209–49. doi: 10.3322/caac.21660, PMID: 33538338

[3] Kohoutova D Banks M Bures J . Advances in the aetiology & Endoscopic detection and management of early gastric cancer. Cancers. (2021) 13:6242. doi: 10.3390/cancers13246242, PMID: 34944861 PMC8699285

[4] Guan WL He Y Xu RH . Gastric cancer treatment: recent progress and future perspectives. J Hematol Oncol. (2023) 16:57. doi: 10.1186/s13045-023-01451-3, PMID: 37245017 PMC10225110

[5] Joshi SS Badgwell BD . Current treatment and recent progress in gastric cancer. Ca-Cancer J Clin. (2021) 71:264–79. doi: 10.3322/caac.21657, PMID: 33592120 PMC9927927

[6] Shapovalov G Ritaine A Skryma R Prevarskaya N . Role of TRP ion channels in cancer and tumorigenesis. Semin Immunopathol. (2016) 38:357–69. doi: 10.1007/s00281-015-0525-1, PMID: 26842901

[7] Mignen O Constantin B Potier-Cartereau M Penna A Gautier M Gueguinou M . Constitutive calcium entry and cancer: updated views and insights. Eur Biophys J Biophy. (2017) 46:395–413. doi: 10.1007/s00249-017-1216-8, PMID: 28516266

[8] Almasi S Sterea AM Fernando W Clements DR Marcato P Hoskin DW . TRPM2 ion channel promotes gastric cancer migration, invasion and tumor growth through the AKT signaling pathway. Sci Rep-UK. (2019) 9:4182. doi: 10.1038/s41598-019-40330-1, PMID: 30862883 PMC6414629

[9] Xie R Xu J Xiao Y Wu J Wan H Tang B . Calcium promotes human gastric cancer via a novel coupling of calcium-sensing receptor and TRPV4 channel. Cancer Res. (2017) 77:6499–512. doi: 10.1158/0008-5472.CAN-17-0360, PMID: 28951460

[10] Ge P Wei L Zhang M Hu B Wang K Li Y . TRPC1/3/6 inhibition attenuates the TGF-beta1-induced epithelial-mesenchymal transition in gastric cancer via the Ras/Raf1/ERK signaling pathway. Cell Biol Int. (2018) 42:975–84. doi: 10.1002/cbin.10963, PMID: 29570903

[11] Ooi CH Ivanova T Wu J Lee M Tan IB Tao J . Oncogenic pathway combinations predict clinical prognosis in gastric cancer. PloS Genet. (2009) 5:e1000676. doi: 10.1371/journal.pgen.1000676, PMID: 19798449 PMC2748685

[12] Wang G Hu N Yang HH Wang L Su H Wang C . Comparison of global gene expression of gastric cardia and noncardia cancers from a high-risk population in China. PloS One. (2013) 8:e63826. doi: 10.1371/journal.pone.0063826, PMID: 23717493 PMC3661768

[13] Lei Z Tan IB Das K Deng N Zouridis H Pattison S . Identification of molecular subtypes of gastric cancer with different responses to PI3-kinase inhibitors and 5-fluorouracil. Gastroenterology. (2013) 145:554–65. doi: 10.1053/j.gastro.2013.05.010, PMID: 23684942

[14] Qian Z Zhu G Tang L Wang M Zhang L Fu J . Whole genome gene copy number profiling of gastric cancer identifies PAK1 and KRAS gene amplification as therapy targets. Gene Chromosome Canc. (2014) 53:883–94. doi: 10.1002/gcc.22196, PMID: 24935174

[15] Cristescu R Lee J Nebozhyn M Kim KM Ting JC Wong SS . Molecular analysis of gastric cancer identifies subtypes associated with distinct clinical outcomes. Nat Med. (2015) 21:449–56. doi: 10.1038/nm.3850, PMID: 25894828

[16] Yoon SJ Park J Shin Y Choi Y Park SW Kang SG . Deconvolution of diffuse gastric cancer and the suppression of CD34 on the BALB/c nude mice model. BMC Cancer. (2020) 20:314. doi: 10.1186/s12885-020-06814-4, PMID: 32293340 PMC7160933

[17] Kang B Camps J Fan B Jiang H Ibrahim MM Hu X . Parallel single-cell and bulk transcriptome analyses reveal key features of the gastric tumor microenvironment. Genome Biol. (2022) 23:265. doi: 10.1186/s13059-022-02828-2, PMID: 36550535 PMC9773611

[18] Colaprico A Silva TC Olsen C Garofano L Cava C Garolini D . TCGAbiolinks: an R/Bioconductor package for integrative analysis of TCGA data. Nucleic Acids Res. (2016) 44:e71. doi: 10.1093/nar/gkv1507, PMID: 26704973 PMC4856967

[19] Kauffmann A Rayner TF Parkinson H Kapushesky M Lukk M Brazma A . Importing ArrayExpress datasets into R/Bioconductor. Bioinformatics. (2009) 25:2092–4. doi: 10.1093/bioinformatics/btp354, PMID: 19505942 PMC2723004

[20] Leek JT Johnson WE Parker HS Jaffe AE Storey JD . The sva package for removing batch effects and other unwanted variation in high-throughput experiments. Bioinformatics. (2012) 28:882–3. doi: 10.1093/bioinformatics/bts034, PMID: 22257669 PMC3307112

[21] Hao Y Stuart T Kowalski MH Choudhary S Hoffman P Hartman A . Dictionary learning for integrative, multimodal and scalable single-cell analysis. Nat Biotechnol. (2024) 42:293–304. doi: 10.1038/s41587-023-01767-y, PMID: 37231261 PMC10928517

[22] Wilkerson MD Hayes DN . ConsensusClusterPlus: a class discovery tool with confidence assessments and item tracking. Bioinformatics. (2010) 26:1572–3. doi: 10.1093/bioinformatics/btq170, PMID: 20427518 PMC2881355

[23] Xu T Le TD Liu L Su N Wang R Sun B . CancerSubtypes: an R/Bioconductor package for molecular cancer subtype identification, validation and visualization. Bioinformatics. (2017) 33:3131–3. doi: 10.1093/bioinformatics/btx378, PMID: 28605519

[24] Ritchie ME Phipson B Wu D Hu Y Law CW Shi W . limma powers differential expression analyses for RNA-sequencing and microarray studies. Nucleic Acids Res. (2015) 43:e47. doi: 10.1093/nar/gkv007, PMID: 25605792 PMC4402510

[25] Zeng D Fang Y Qiu W Luo P Wang S Shen R . Enhancing immuno-oncology investigations through multidimensional decoding of tumor microenvironment with IOBR 2.0. Cell Rep Methods. (2024) 4:100910. doi: 10.1016/j.crmeth.2024.100910, PMID: 39626665 PMC11704618

[26] Krug K Mertins P Zhang B Hornbeck P Raju R Ahmad R . A curated resource for phosphosite-specific signature analysis. Mol Cell Proteomics. (2019) 18:576–93. doi: 10.1074/mcp.TIR118.000943, PMID: 30563849 PMC6398202

[27] Langfelder P Horvath S . WGCNA: an R package for weighted correlation network analysis. BMC Bioinf. (2008) 9:559. doi: 10.1186/1471-2105-9-559, PMID: 19114008 PMC2631488

[28] Butler A Hoffman P Smibert P Papalexi E Satija R . Integrating single-cell transcriptomic data across different conditions, technologies, and species. Nat Biotechnol. (2018) 36:411–20. doi: 10.1038/nbt.4096, PMID: 29608179 PMC6700744

[29] Zhang X Lan Y Xu J Quan F Zhao E Deng C . CellMarker: a manually curated resource of cell markers in human and mouse. Nucleic Acids Res. (2019) 47:D721–8. doi: 10.1093/nar/gky900, PMID: 30289549 PMC6323899

[30] Wang K Wang M Li Z Hu B Wu J Yuan Z . An antigen processing and presentation signature for prognostic evaluation and immunotherapy selection in advanced gastric cancer. Front Immunol. (2022) 13:992060. doi: 10.3389/fimmu.2022.992060, PMID: 36311733 PMC9615473

[31] Luo C Zhang R Guo R Wu L Xue T He Y . Integrated computational analysis identifies therapeutic targets with dual action in cancer cells and T?cells. IMMUNITY. (2025) 58:745–65. doi: 10.1016/j.immuni.2025.02.007, PMID: 40023158

[32] Deng C Li ZX Xie CJ Zhang QL Hu BS Wang MD . Pan-cancer analysis of CDKN2A alterations identifies a subset of gastric cancer with a cold tumor immune microenvironment. Hum Genomics. (2024) 18:55. doi: 10.1186/s40246-024-00615-7, PMID: 38822443 PMC11143690

[33] Yan J Chen D Ye Z Zhu X Li X Jiao H . Molecular mechanisms and therapeutic significance of Tryptophan Metabolism and signaling in cancer. Mol Cancer. (2024) 23:241. doi: 10.1186/s12943-024-02164-y, PMID: 39472902 PMC11523861

[34] Koivisto AP Belvisi MG Gaudet R Szallasi A . Advances in TRP channel drug discovery: from target validation to clinical studies. Nat Rev Drug Discov. (2022) 21:41–59. doi: 10.1038/s41573-021-00268-4, PMID: 34526696 PMC8442523

[35] Pan T Gao Y Xu G Zhou P Li S Guo J . Pan-cancer analyses reveal the genetic and pharmacogenomic landscape of transient receptor potential channels. NPJ Genom Med. (2022) 7:32. doi: 10.1038/s41525-022-00304-1, PMID: 35614079 PMC9132893

[36] Shi Q Xue C Zeng Y Yuan X Chu Q Jiang S . Notch signaling pathway in cancer: from mechanistic insights to targeted therapies. Signal Transduct Tar. (2024) 9:128. doi: 10.1038/s41392-024-01828-x, PMID: 38797752 PMC11128457

[37] Cao Z An L Han Y Jiao S Zhou Z . The Hippo signaling pathway in gastric cancer. Acta Bioch Bioph Sin. (2023) 55:893–903. doi: 10.3724/abbs.2023038, PMID: 36924251 PMC10326414

[38] Fattahi S Amjadi-Moheb F Tabaripour R Ashrafi GH Akhavan-Niaki H . PI3K/AKT/mTOR signaling in gastric cancer: Epigenetics and beyond. Life Sci. (2020) 262:118513. doi: 10.1016/j.lfs.2020.118513, PMID: 33011222

[39] Wang X Li N Liu YH Wu J Liu QG Niu JB . Targeting focal adhesion kinase (FAK) in cancer therapy: A recent update on inhibitors and PROTAC degraders. Eur J Med Chem. (2024) 276:116678. doi: 10.1016/j.ejmech.2024.116678, PMID: 39029337

[40] Kim ST Cristescu R Bass AJ Kim KM Odegaard JI Kim K . Comprehensive molecular characterization of clinical responses to PD-1 inhibition in metastatic gastric cancer. Nat Med. (2018) 24:1449–58. doi: 10.1038/s41591-018-0101-z, PMID: 30013197

[41] Nie K Li J Peng L Zhang M Huang W . Pan-cancer analysis of the characteristics of LY96 in prognosis and immunotherapy across human cancer. Front Mol Biosci. (2022) 9:837393. doi: 10.3389/fmolb.2022.837393, PMID: 35647025 PMC9130738

[42] Wang H Zhang B Wang X Mao J Li W Sun Y . TRPV4 overexpression promotes metastasis through epithelial-mesenchymal transition in gastric cancer and correlates with poor prognosis. Oncotargets Ther. (2020) 13:8383–94. doi: 10.2147/OTT.S256918, PMID: 32943876 PMC7468412

[43] Siveen KS Nizamuddin PB Uddin S Al-Thani M Frenneaux MP Janahi IA . TRPV2: A cancer biomarker and potential therapeutic target. Dis Markers. (2020) 2020:8892312. doi: 10.1155/2020/8892312, PMID: 33376561 PMC7746447

[44] Stoklosa P Borgstrom A Kappel S Peinelt C . TRP channels in digestive tract cancers. Int J Mol Sci. (2020) 21:1877. doi: 10.3390/ijms21051877, PMID: 32182937 PMC7084354

[45] Yi H Lin Y Wang X Mao Y . Pan-cancer analysis of TRPV2 identifies its potential role as a prognostic and immunologic biomarker in oesophageal cancer. Brit J Cancer. (2023) 129:567–9. doi: 10.1038/s41416-023-02302-1, PMID: 37311976 PMC10421898

[46] Gao Y Liao P . TRPM4 channel and cancer. Cancer Lett. (2019) 454:66–9. doi: 10.1016/j.canlet.2019.04.012, PMID: 30980865

[47] Liu Q Hu M Li S Zhang X Zhang R Lyu H . TRPM channels in human cancers: regulatory mechanism and therapeutic prospects. biomark Res. (2024) 12:152. doi: 10.1186/s40364-024-00699-2, PMID: 39633507 PMC11616203

[48] Lee C Lee JB Park SJ Che J Kwon WS Kim HS . Second-line chemoimmunotherapy with nivolumab and paclitaxel in immune-related biomarker-enriched advanced gastric cancer: a multicenter phase Ib/II study. Gastric cancer: Off J Int Gastric Cancer Assoc Japanese Gastric Cancer Assoc. (2024) 27:118–30. doi: 10.1007/s10120-023-01435-9, PMID: 37906316

[49] Fu S Hirte H Welch S Ilenchuk TT Lutes T Rice C . First-in-human phase I study of SOR-C13, a TRPV6 calcium channel inhibitor, in patients with advanced solid tumors. Invest New Drug. (2017) 35:324–33. doi: 10.1007/s10637-017-0438-z, PMID: 28150073 PMC5418314

[50] Ardura JA Álvarez-Carrión L Gutiérrez-Rojas I Alonso V . Role of calcium signaling in prostate cancer progression: effects on cancer hallmarks and bone metastatic mechanisms. Cancers. (2020) 12:1071. doi: 10.3390/cancers12051071, PMID: 32344908 PMC7281772

[51] Coroller TP Agrawal V Narayan V Hou Y Grossmann P Lee SW . Radiomic phenotype features predict pathological response in non-small cell lung cancer. Radiotherapy Oncol J Eur Soc Ther Radiol Oncol. (2016) 119:480–6. doi: 10.1016/j.radonc.2016.04.004, PMID: 27085484 PMC4930885

[52] Jin Y Ide H Nagata M Kobayashi T Lu J Ikehata Y . Pre-treatment monocytic myeloid-derived suppressor cells as predictive biomarkers for immune checkpoint inhibitor response in clear cell renal cell carcinoma. Front Immunol. (2025) 16:1641383. doi: 10.3389/fimmu.2025.1641383, PMID: 40918141 PMC12408328

[53] Xu Q Wang Z Huang S Shi J Zhu Y Pang H . New prognostic features and personalized treatment strategies of mitochondrial related genes in colorectal cancer patients. Front Pharmacol. (2025) 16:1540767. doi: 10.3389/fphar.2025.1540767, PMID: 40290445 PMC12023264

