# Supplementary figures and images for: TRP channel expression patterns define molecular subtypes, prognosis, and therapeutic targets in gastric cancer
Source: Front Immunol. 2026 Mar 5;17:1752001. doi: 10.3389/fimmu.2026.1752001 (PMC12999853; doi:10.3389/fimmu.2026.1752001)

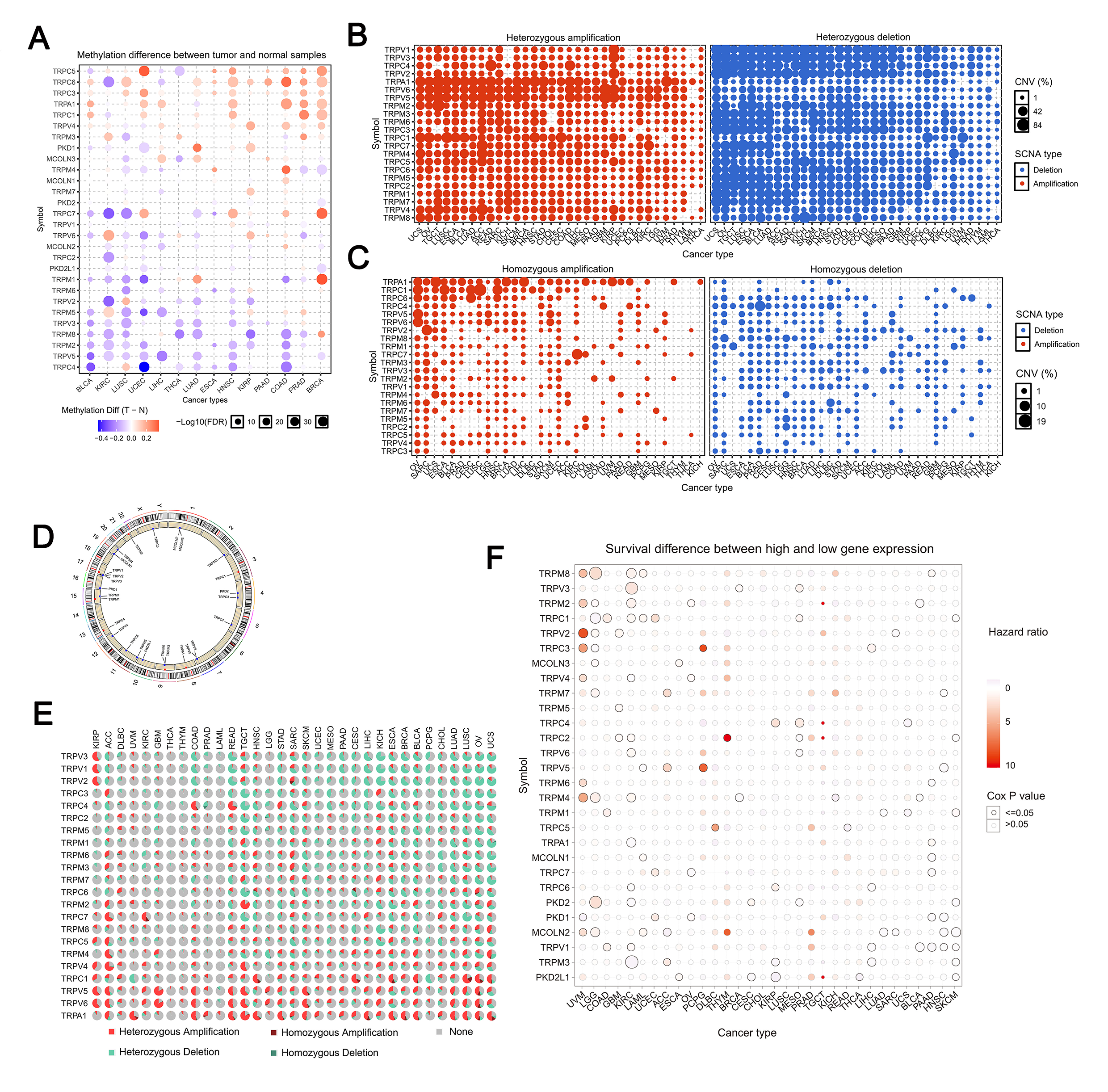

Supplement: Supplementary Figure 1 — Genomic alterations and survival associations of TRP channel regulators across cancer types. (A) Bubble chart showing the differences in methylation levels of 28 TRP channel regulators between tumor and normal tissues across cancers. Methylation Diff (T−N): the mean methylation level of tumor samples minus that of normal samples. (B) The heterozygous CNV bubble chart displays the percentages of heterozygous CNV amplifications and deletions for each gene across cancers. (C) The heterozygous CNV bubble chart illustrates the percentages of homozygous CNV amplifications and deletions for each gene across cancers. (D) The location of CNV alterations for the 28 TRP channel regulators on the 23 chromosomes in the STAD cohort. (E) CNV pie charts depict the distribution of CNV categories (heterozygous and homozygous amplification/deletion) for each gene in selected cancers. Each pie represents the proportion of CNV types for TRP channel regulators in a specific cancer type, with different colors corresponding to distinct CNV types. None: no CNV detected. (F) Bubble chart presenting survival differences between patients with high and low expression of TRP channel regulators across cancers. Survival analysis was performed using the Cox proportional hazards model to calculate HRs for each gene, followed by Kaplan–Meier survival analysis with a log-rank test. Bubbles on the solid line indicating statistical significance (P ≤ 0.05). [file Image1.tif]

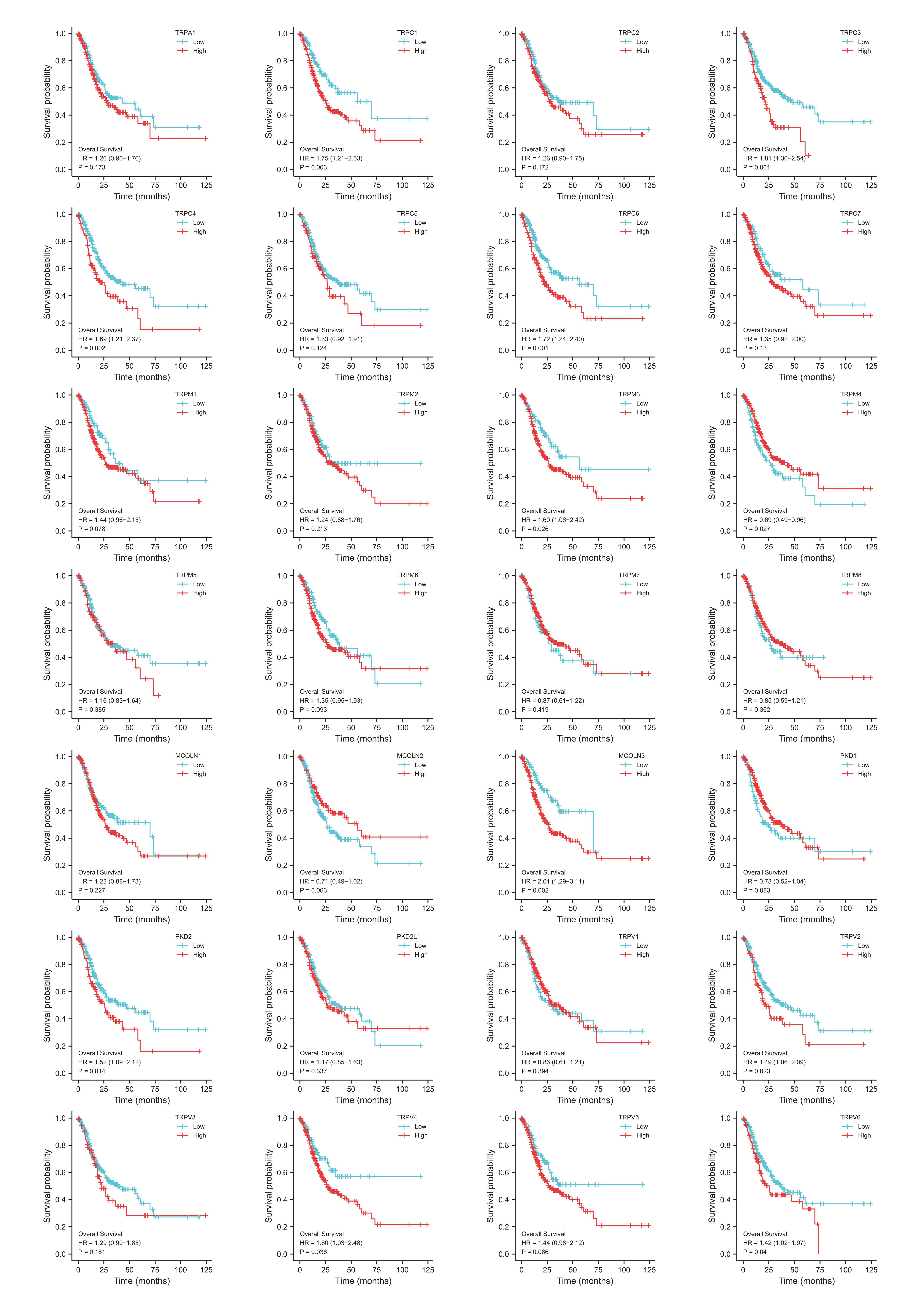

Supplement: Supplementary Figure 2 — The survival differences between the high- and low-expression groups of the 28 TRP channel regulators were determined using the maximum Youden index as the cut-off value. [file Image2.tif]

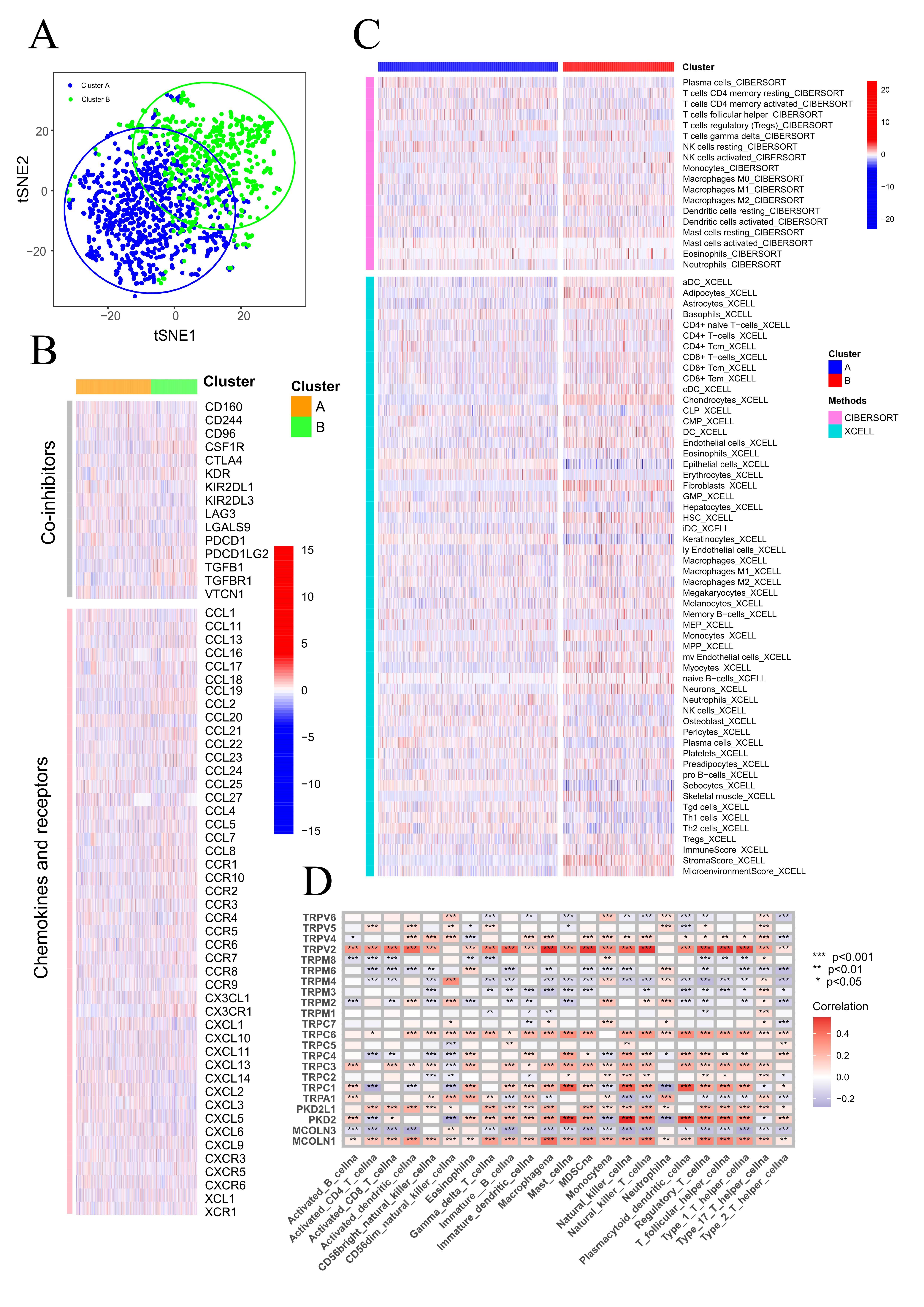

Supplement: Supplementary Figure 4 — TRP subtypes and immune landscape in GC. (A) The tSNE plot demonstrates that the two TRP subtypes are characterized by distinct expression levels of the 22 TRP channel regulators. (B) Comparison of key tumor immune function genes between two TRP subtypes. (C) Distribution of immune cell infiltration and immune scores between two TRP subtypes. (D) Correlation analysis of immune cell infiltration and immune function scores with the 22 TRP channel regulators based on ssGSEA analysis. The correlation analysis was conducted using the two-tailed Spearman correlation. [file Image4.jpeg]

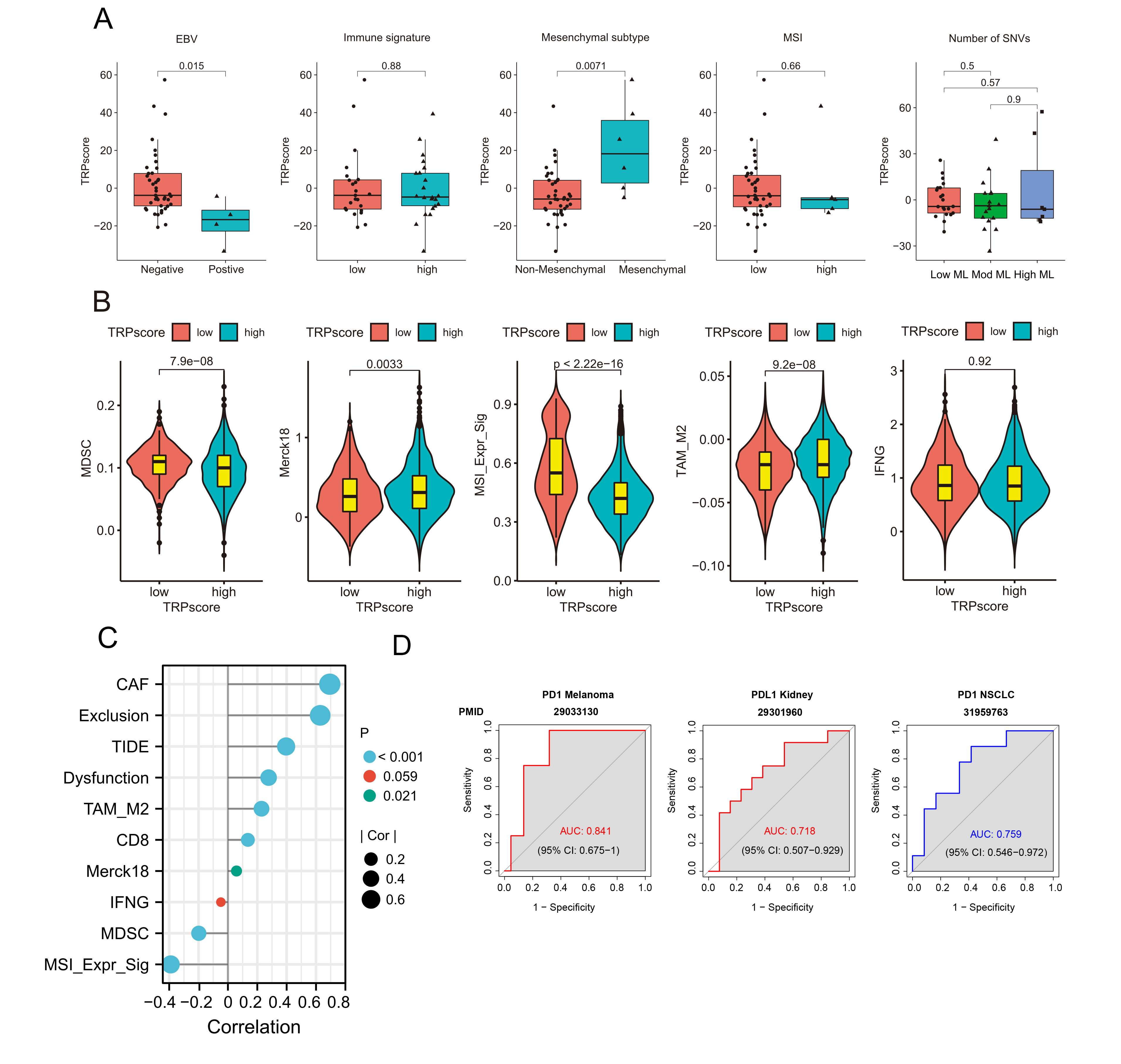

Supplement: Supplementary Figure 7 — Evaluation of immune therapy response based on TRPscore. (A) Distribution of TRPscore across EBV, Immune Signature, Mesenchymal, MSI, and Number of SNVs in GC. (B) Comparison of MDSC, Merck18, MSI, TAM_M2, and IFNG between high-TRPscore and low-TRPscore groups in 1544 samples. (C) Correlation analysis of TRPscore with TIDE-related predictive indicators. (D) Cross-cancer validation of TRPscore for predicting immune checkpoint blockade response. Receiver operating characteristic (ROC) curves for three independent cohorts: renal cell carcinoma, melanoma, and non-small cell lung cancer. [file Image7.jpeg]
